# High-precision estimation of emitter positions using Bayesian grouping of localizations

**DOI:** 10.1038/s41467-022-34894-2

**Published:** 2022-11-22

**Authors:** Mohamadreza Fazel, Michael J. Wester, David J. Schodt, Sebastian Restrepo Cruz, Sebastian Strauss, Florian Schueder, Thomas Schlichthaerle, Jennifer M. Gillette, Diane S. Lidke, Bernd Rieger, Ralf Jungmann, Keith A. Lidke

**Affiliations:** 1 Department of Physics and Astronomy, University of New Mexico, Albuquerque, NM USA; 2 Department of Mathematics and Statistics, University of New Mexico, Albuquerque, NM USA; 3 Department of Pathology, University of New Mexico Health Science Center, Albuquerque, NM USA; 4 Department of Physics and Center for Nanoscience, Ludwig Maximilian University, Munich, Germany; 5 Max Planck Institute of Biochemistry, Martinsried, Germany; 6 Comprehensive Cancer Center, University of New Mexico, Albuquerque, NM USA; 7 Department of Imaging Physics, Delft University of Technology, Delft, the Netherlands

**Keywords:** Biological fluorescence, Single-molecule biophysics, Super-resolution microscopy, Fluorescence imaging

## Abstract

Single-molecule localization microscopy super-resolution methods rely on stochastic blinking/binding events, which often occur multiple times from each emitter over the course of data acquisition. Typically, the blinking/binding events from each emitter are treated as independent events, without an attempt to assign them to a particular emitter. Here, we describe a Bayesian method of inferring the positions of the tagged molecules by exploring the possible grouping and combination of localizations from multiple blinking/binding events. The results are position estimates of the tagged molecules that have improved localization precision and facilitate nanoscale structural insights. The Bayesian framework uses the localization precisions to learn the statistical distribution of the number of blinking/binding events per emitter and infer the number and position of emitters. We demonstrate the method on a range of synthetic data with various emitter densities, DNA origami constructs and biological structures using DNA-PAINT and dSTORM data. We show that under some experimental conditions it is possible to achieve sub-nanometer precision.

## Abstract (web summary)

Single-molecule localization microscopy relies on stochastic blinking events, treated as independent events without assignment to a particular emitter. Here, BaGoL takes low precision localizations generated from multiple emitter blinkings during DNAPAINT and dSTORM and finds the underlying emitter positions with high precision.

## Introduction

Fluorescence super-resolution microscopy methods exploit the independent behavior of fluorescent molecules to circumvent the diffraction limit^1,2^. Single-molecule localization microscopy (SMLM) methods combine the independent and sparse blinking of fluorophores with direct inference of fluorophore’s locations^3–6^. The SMLM methods of (d)STORM^3,6^ and DNA-PAINT^7^ often undergo multiple blinking/binding events from each fluorophore and these blinking events are randomly spaced temporally throughout the data collection. Throughout the manuscript, we refer to a ‘localization’ as the estimate of the position of the fluorophore from a single blinking/binding event and an ‘emitter’ as the fluorescently tagged molecule. These localizations can be used to reconstruct high-resolution images of the underlying biological structures^8^. Yet, the presence of multiple low precision individual localizations per emitter hinders quantification of the underlying biological structures required for understanding biology on nanometer scale. For instance, better precision directly improves estimates of the position and spacing between molecules, which is necessary for understanding the structure and function of macromolecular complexes^9^ and signaling clusters^10–12^.

Each of multiple individual localizations generated from an emitter during SMLM methods carries with it information that can be employed to estimate the underlying emitter position with high precision, scaling as approximately 1/sqrt(*λ*) where *λ* is the number of repeat blinking/binding events (Supplementary Note 4). However, in practice, emitters exist in close proximity (often a few nanometers apart) leading to dense areas containing many localizations with low precisions. This in turn introduces considerable uncertainty in estimating the underlying emitter positions and allocation of sub-groups of localizations to these emitters. Therefore, to incorporate all the existing sources of uncertainties into the problem, we adopt a Bayesian framework to explore all the possible grouping configurations for a set of given localizations in order to make inference about the number and positions of emitters.

There have been a few attempts to correct for multiple localizations per emitter, however, these methods are specialized to only dSTORM and PALM^13,14^ as they rely on photobleaching to enumerate emitters. These methods use maximum likelihood estimation which does not propagate uncertainty from all the existing sources, e.g., uncertainty in the number of emitters and localization precisions, throughout the problem. Here, we advance the state-of-the-art by lifting the photobleaching requirement using a Bayesian Grouping of Localizations (BaGoL) to deal with both dSTORM and DNA-PAINT data while rigorously propagating uncertainty from all the existing sources. BaGoL is also capable of correcting for residual drift in the input localizations and accomplishes sub-nanometer precisions under dense labeling conditions.

Here, we describe and demonstrate the BaGoL method. BaGoL uses the observed localizations and their uncertainties to explore the possible number of emitters (*K*), allocation of localizations to emitters (*Z*), the distribution of the number of localizations per emitter (*ξ*), and uses this information to make estimates of emitter positions (*θ*), often with substantially improved precision. In the Bayesian paradigm, we make inference about the set of unknown parameters (*K*, *Z*, *ξ*, *θ*) by taking samples from the posterior probability distribution of these parameters^15^. The posterior probability distribution is proportional to the product of the likelihood model and prior distributions over the unknown parameters (Supplementary Note 1). In practice, the BaGoL’s posterior has a complicated form with a variable number of parameters, which stems from an unknown number of emitters *K*, and cannot be directly sampled. Therefore, BaGoL uses Reversible Jump Markov Chain Monte Carlo (RJMCMC)^16^ to draw samples from the posterior to facilitate inference of the number of parameters, i.e., model.

While Markov Chain Monte Carlo (MCMC) is confined to problems with a fixed number of parameters (fixed model), RJMCMC makes inferences about the number of parameters (model inference) as well as the parameters themselves. To do so, RJMCMC constructs a chain of samples using an extension to the Metropolis-Hasting algorithm^17–19^ to vary the model throughout the chain. The returned chain can then be used to find the probability distributions of both the models and the parameters within each model. Our method therefore uses RJMCMC to vary the number of emitters to explore the possibility of different models and can be used to make a weighted average over all models or to find the most probable model (Supplementary Note 2).

**Fig. 1:**
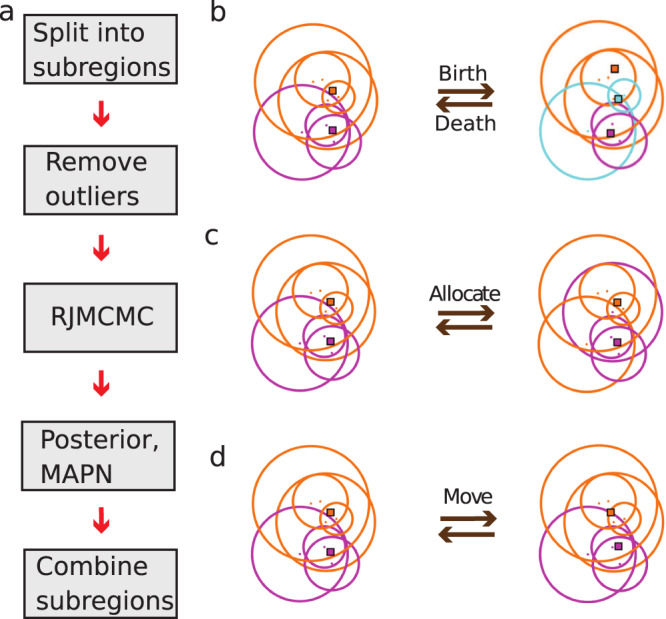
Bayesian grouping of localizations concept and data flow. Circles are centered on given localizations with radii equal to two times the corresponding localization precisions. Colors represent localization allocations to emitters. Squares show emitters. **a** The data flow. The RJMCMC step is illustrated in further detail in panels **b**–**d**. **b** From left to right, addition of a new emitter (blue) in a random location is proposed via a *Birth* jump. From right to left, an existing emitter is picked randomly and is eliminated from the model via a *Death* jump. **c** Localizations are redistributed across the emitters via an *Allocation* jump while emitter positions are fixed. **d** Given a fixed set of allocations, all emitter positions are updated in a *Move* jump.

The input to the BaGoL algorithm is a set of localizations, uncertainties, and time stamps generated by a traditional SMLM analysis^20^. If there is a priori knowledge of the probability distribution for the number of blinking/binding events of a single label, *ξ*, such as from a dedicated control experiment where the sample is known to be at low labeling fraction^21^, this can be given to the algorithm. Otherwise, this distribution can be directly learned from the data. The complete algorithm consists of several steps (Fig. 1, Supplementary Notes 2–3): (1) Splitting the set of coordinates into smaller subregions to speed up the analysis; (2) Removing outliers using a filtering step; (3) The RJMCMC algorithm; (4) Generating the model with *maximum a posteriori* number of emitters (MAPN) (i.e., the most repeated/probable model) and posterior probability results; and (5) Stitching back together the results of the subregions. We briefly describe each step below whereas mathematical details can be found in the Supplementary Information.

1. To speed up the calculations, the data set is split into sub-regions with small overlaps that are used to account for edge effects. The size of subregions is typically adjusted such that subregion size is inversely correlated with the data density. There is no restriction on the size of the subregions other than its effect on computational speed. We recommend using subregions with not more than a few thousand localizations.
2. Localizations that were not generated from a single emitter in the sample are considered outliers which can negatively affect the performance of the algorithm and are therefore removed before analysis, Supplementary Fig. 17. These outliers may arise, for example, from fitting two closely spaced emitters as a single emitter or from non-specific binding. To eliminate these outliers, we include two types of optional pre-analysis filtering methods: (a) localizations with an intensity higher than ~2 times the mean are removed to prevent incorrect, but high-precision localizations from entering the analysis^22^; (b) in DNA-PAINT data where many localizations per emitter are expected but there is the possibility of spurious non-specific binding events, localizations that don’t have many nearby neighbors are removed. We call this procedure the NND filter which is further detailed in the Methods section and Supplementary Fig. 17, where NND stands for nearest neighbor distribution. We emphasize that the NND filter must not be used for dSTORM data where it may be possible to have only one localization per emitter.
3. The core RJMCMC step constructs a chain of samples by iteratively sweeping the parameter set including: the number of emitters, *K*; the emitters’ positions and drifts, denoted by *θ* = {*μ*_*1*_*,a*_*1*_.._,_*μ*_*K*_*,a*_*K*_} where *μ*_*k*_ is the location of the *k*th emitter and *a*_*k*_ models a linear drift term of the *k*th emitter; and the allocation of the *N* localizations to *K* underlying emitters, *Z* (Supplementary Movie 1). In each iteration, a parameter is selected at random and explored by proposing a new value by a small jump in the current value. BaGoL, uses four jump types to update the parameter values illustrated in Fig. 1. *Move* selects new values of *θ* by directly drawing samples from the posterior given the current values for the rest of parameters. *Allocate* redistributes all localizations across the *K* emitters while assuming the remaining parameters stay fixed. *Birth* adds a new emitter to the current model and redistributes the localizations. *Death* removes one of the emitters and redistributes the localizations. Here, the *Move* jump is always accepted by virtue of taking direct samples from the posterior, whereas the other jumps are accepted or rejected with a probability *A* as given by the rules of RJMCMC (Supplementary Note 2). During the described RJMCMC iteration, the localization per emitter distribution is used as a prior distribution. This distribution itself, parameterized by *ξ*, is explored using a hierarchical Bayes approach, where information from data and the current state of the chain is propagated to *ξ* through the number of emitters, *K*; Supplementary Fig. 18. To facilitate mixing of the chain, multiple RJMCMC steps are taken in between each MCMC step that proposes a new *ξ*. The complete formalism is described in Supplementary Note 1–2. Finally, the constructed chain of samples is returned and used for the subsequent numerical analyses.
4. BaGoL uses the chain of samples from the RJMCMC step to generate two output images: posterior and MAPN images. The posterior image is a weighted average over all the possible models of emitter positions, generated by a histogram-type 2D image of the emitter positions stored in the chain. Moreover, the model that has the most repeated/probable model is extracted from the chain and is used to generate a MAPN image in the same manner as the posterior probability image. The extracted model is also used to calculate the MAPN emitter coordinates and their uncertainties using k-means clustering of the distribution of emitter positions in the chain^23^; Supplementary Note 3. For the posterior image and resulting MAPN coordinates, emitter positions that fall into the overlapping regions are removed.
5. The posterior images for the subregions are combined to give a single BaGoL reconstructed posterior image of the entire region. The MAPN image of the entire region is also constructed by combining the MAPN images of subregions.

## Results

**Fig. 2:**
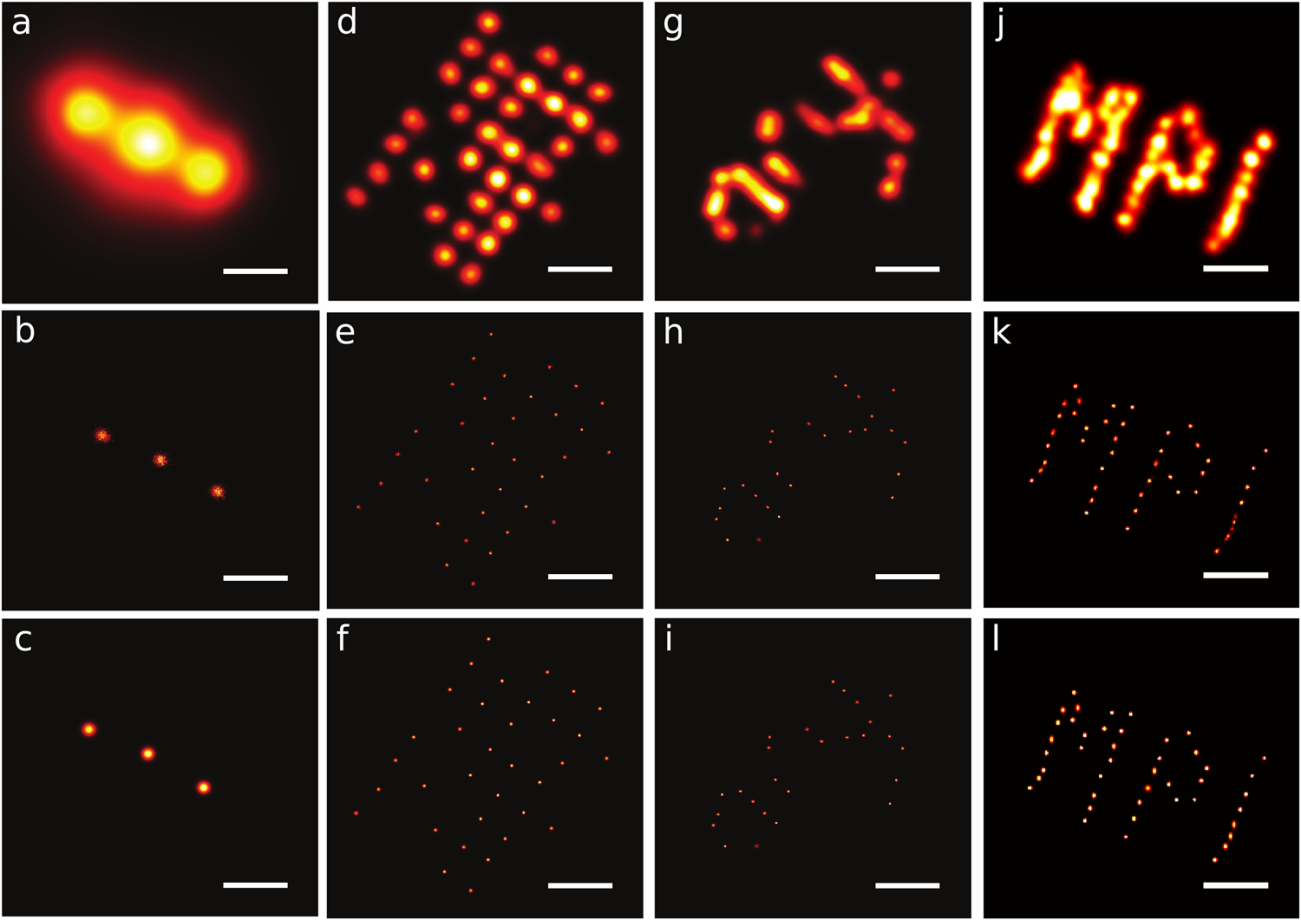
Bayesian grouping of localizations applied to various structures imaged with DNA-PAINT. Row 1: Traditional SR analysis with each localization represented by a Gaussian blob of the size of its localization precision. Row 2: Posterior probability image of the chain from BaGoL including all the proposed models. Row 3: The image from the model with the most likely number of emitters (MAPN). **a**–**c** Gattaquant DNA rulers with 20 nm spacing between docking strands. The shown images were selected from the BaGoL results of 50 similar structures; **d**–**f** DNA-origami grid with 10 nm spacing between docking strands. The shown images were selected from the BaGoL results of 170 similar structures; **g**–**i** TUD DNA origami with 5 nm spacing between docking strands. The shown images were selected from the BaGoL results of 170 similar structures (see Supplementary Movie 2); **j**–**l** MPI DNA origami with 5 nm spacing between docking strands. The shown images were selected from the BaGoL results of four similar structures. The source data is provided within the paper. The scale bars are 20 nm.

**Fig. 3:**
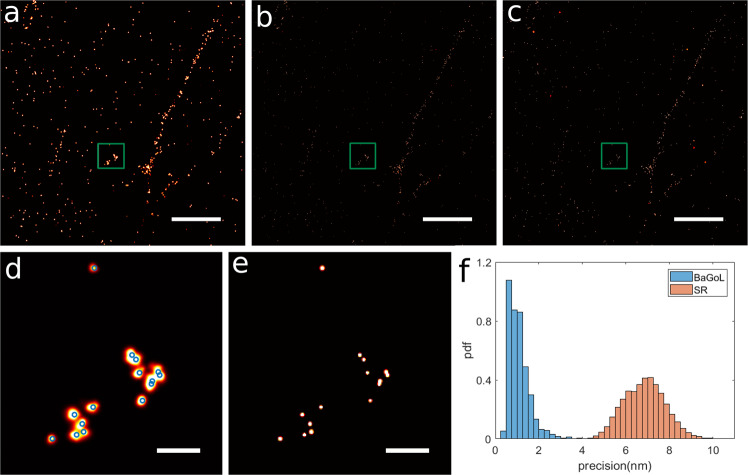
BaGoL applied to the focal adhesion protein kindlin. The protein kindlin-GFP was visualized using a DNA-labeled GFP nanobody and imaged via DNA-PAINT. **a** 5 × 5 μm^2^ region of traditional super-resolution image. **b** BaGoL posterior image of panel **a**. **c** BaGoL MAPN image of panel **a**. **d** Zoom-in of the green box in panel **a**. Blue circles represent the found emitter coordinates by BaGoL. **e** Zoom-in of the green box in panel **c**. **f** Histograms of the localization precisions from SR data shown in brown (input to BaGoL), and the improved precisions from BaGoL shown in blue. BaGoL was applied to the focal adhesion protein kindlin data for three similar regions. Scale bars in the top and bottom rows are 1000 and 100 nm, respectively.

**Fig. 4:**
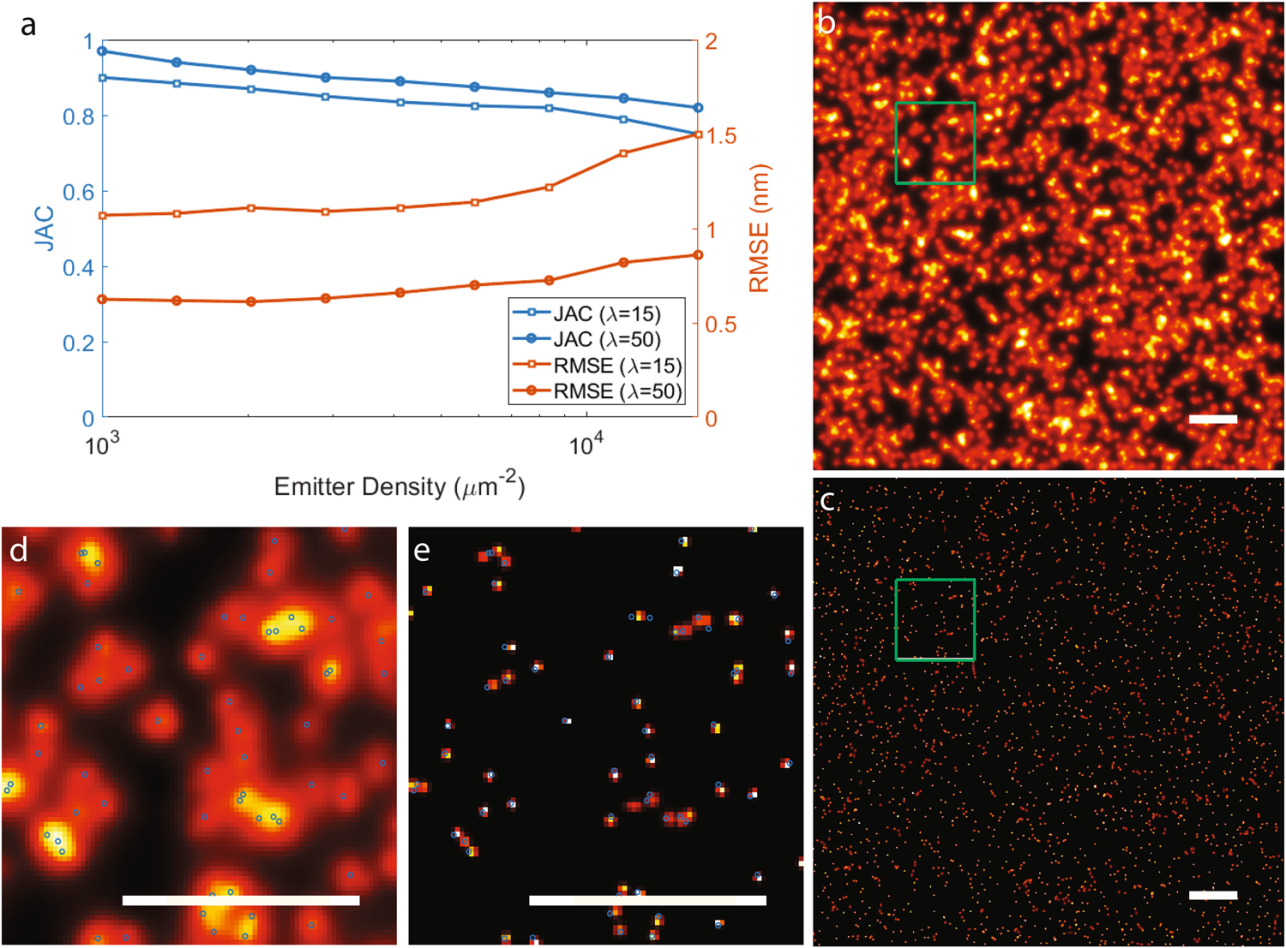
Jaccard index (JAC) and root mean square error (RMSE). JAC and RMSE were calculated for nine logarithmically spaced concentrations starting from 1000 emitters per μm^2^ to 17000 emitters per μm^2^ corresponding to average emitter separations ranging from 15.8 to 3.8 nm. **a** JAC and RMSE was calculated by averaging over outcomes of five simulated data sets for each concentration. **b** An example of simulated SR-data at a concentration of 10,000 emitters per μm^2^ (average emitter separation of 5 nm) with *λ* = 50 simulated over an area of 500 × 500 nm^2^. **c** MAPN results of panel **b** where each found emitter is presented by a Gaussian blob centered at the found location and a width similar to the corresponding localization precision. **d** Zoom-in of the green box in panel **b**. **e** Zoom-in of the green box in panel **c**. The BaGoL analysis in panel b-e was repeated for five similar data sets. Details of the data simulation and analysis are described in the “Methods” section. The blue circles represent the ground truth emitter locations. Scale bars are 50 nm.

We benchmark BaGoL using a range of experimental and realistic synthetic data from both DNA-PAINT and dSTORM methods. First, we show that BaGoL facilitates inspection of structures by combining localizations to improve precisions using experimental DNA-PAINT data from DNA origami structures with emitter separations varying from 20 nm to the challenging case of 5 nm, as well as biological samples; see Figs. 2–3. We further show that our method accomplishes sub-nanometer precisions for multiple DNA origami structures in Fig. 2. Moreover, we use synthetic DNA-PAINT data to benchmark various aspects of our method under different conditions including: (1) different emitter separations/densities (Fig. 4 and Supplementary Figs. 1–7); (2) different number of localizations per emitter (Fig. 4 and Supplementary Figs. 2–7); (3) presence of drift (Supplementary Fig. 8). Using synthetic data, we also show that BaGoL: (1) can improve resolutions for structures with tightly spaced emitters by improving precisions (Supplementary Fig. 9); (2) multiple subsequent applications of BaGoL on data from identical DNA origamis attains accurate geometry of structures with very high precisions (Supplementary Figs. 10–11). For example, the average accuracy of the predicted positions by BaGoL in Supplementary Fig. 10 is <1 nm; (3) outperforms other existing grouping techniques (Supplementary Fig. 12). Next, we show that BaGoL assists examination of biological structures and inspection of the spatial distribution of molecules using dSTORM data, although it does not exhibit much improvement in precisions due to small number of localizations per emitter (Supplementary Figs. 13–15). To do so, we use a range of biological data from microtubules, CD82, a scaffolding protein found at the plasma membrane, and labeled EGF bound to EGFR (Supplementary Figs. 13–14). Furthermore, we demonstrate BaGoL’s performance using synthetic dSTORM data generated using different emitter separations ranging from 5 to 15 nm (Supplementary Fig. 15). In addition, we compared BaGoL’s performance for synthetic dSTORM data with the DDC algorithm that employs pairwise distance in conjunction with photobleaching to correct for multiple blinking events^13^ (Supplementary Fig. 16). In what follows, we describe our results in more detail.

We begin with Fig. 2, showing results for experimental data collected using various DNA origami structures with decreasing spacing between docking strands from left to right. In Fig. 2a–c we first show the results of BaGoL applied to DNA-PAINT data collected from commercially available 20 nm spaced DNA origami rulers that are intended to be used as test structures. The BaGoL analysis clearly improves upon the traditional SR result and resolves the 20 nm spacing of the ruler with a reported precision of about 1.2 nm (Supplementary Fig. 11). We averaged multiple rulers and applied BaGoL to the combined MAPN results over multiple structures (Supplementary Fig. 11), which gives a ruler separation of 20.7 nm which is consistent with the manufacturer’s specification of 20 nm. Data in Fig. 2d–i was originally collected and used to demonstrate a template-free particle averaging algorithm for SMLM data^24^ whereas the data in Fig. 2j–l uses an improved sequence design^25^ to increase the number of localizations per emitter. The emitter positions in the 10 nm spaced arrays in Fig. 2d–f are clearly resolved and have a sub-nanometer precision of 0.32 nm on average from BaGoL. The TUD and MPI origami in Fig. 2g–l are also resolved with mean precisions of 0.25 and 0.70 nm (sub-nanometer precision) from BaGoL, respectively. In all cases, BaGoL improves upon the traditional SR analysis and reveals the underlying origami structures. Particularly, BaGoL reveals accurate geometry of the TUD and MPI structures (Fig. 2I, l), which have 5 nm spacing between adjacent docking strands in an overall densely labeled local area (Fig. 2g, j).

In Fig. 3, we used BaGoL to analyze DNA-PAINT data of focal adhesion formation and visualized kindlin-2-GFP with a DNA-labeled GFP nanobody^26^. Figure 3a–c represents the conventional super-resolution image with multiple localizations per emitter, posterior image of BaGoL, and MAPN image from BaGoL, respectively. Figure 3d shows a zoomed-in region of the super-resolution image where the blue circles indicate the found MAPN emitter positions by BaGoL. Figure 3e shows the corresponding MAPN images with improved precisions. Figure 3f depicts histograms of localization precisions before and after applying BaGoL showing a factor ~6 improvement in precisions. Here, BaGoL provides a single position for each emitter with high precision (MAPN emitter positions) allowing follow-up quantitative analysis.

We additionally used synthetic data to assess the performance of BaGoL under a wide range of potential imaging conditions. To assess high labeling density conditions with an unknown distribution of localizations per emitter, we used DNA-PAINT simulations with 50 and 15 localizations per emitter on average (*λ* = 50, 15) to build a plot of Jaccard Index (JAC) and root mean square error (RMSE) vs density, Fig. 4a. We used the BaGoL’s MAPN output for quantification and a definition of JAC where a true position and MAPN coordinate are considered a matched pair for the JAC calculation if the true position falls within the 3 sigma precision radius of the MAPN position (see the Methods section). The RMSE was calculated by finding the mean of the differences between the matched pairs in sets of true and found positions. We simulated 5 data sets for every condition and plotted their average of JAC and RMSE in Fig. 4a. As shown in Fig. 4a, BaGoL maintains a ~83% JAC with a RMSE of <1 nm up to 17,000 emitters per μm^2^ (corresponding to an average nearest neighbor distance of 3.8 nm) for *λ* = 50. As expected, the JAC and RMSE results are correlated with the average number of localizations per emitter (*λ*) and they are, respectively, ~75% and 1.5 nm for *λ* = 15 and a density of 17,000 emitters per μm^2^. To show an example of the simulated data used in our calculations of JAC and RMSE, we depicted a simulated data set with 10,000 emitters per μm^2^ (corresponding to an average nearest neighbor distance of 5 nm) and the resulting MAPN coordinates from BaGoL in Fig. 4b, c. Figure 4d, e, respectively, shows zoomed-in images of the green boxes in Fig. 4b, c. The true emitter positions are shown with blue circles in Fig. 4e to compare them with the found positions by BaGoL represented by Gaussian blobs. The sigma of Gaussian blobs are equal to the resulting improved localization precisions from BaGoL.

To assess BaGoL’s potential performance with two closely spaced emitters (two-point resolution), we simulated two emitters with separations from 1 to 10 nm (Supplementary Fig. 1). In all cases, BaGoL gives an improved representation of the true emitter positions as compared to that seen in the traditional SR reconstruction. At 1 nm separation, the correct number of emitters is estimated but the position uncertainty is the same scale as the separation and the two emitters are not resolved. At 2 nm separation, the two emitters are resolved with the reported uncertainty matching well with the observed deviation from the true positions. At larger separations, the precision and accuracy improve, which can be explained by less uncertainty in allocations of localizations to emitters.

In Supplementary Fig. 2–7, we used small synthetic multimeric (8-mer) structures to evaluate and illustrate the impact of emitter densities and number of localizations per emitter on the performance of BaGoL including: (1) deducing the underlying structures; (2) estimation of the number of emitters (Supplementary Fig. 5); (3) improvement in precisions, Supplementary Fig. 6; and (4) accuracy of the estimated emitter positions, Supplementary Fig. 7. Supplementary Figs. 2–4 show the traditional SR reconstruction, the BaGoL posterior image, and the BaGoL MAPN image, respectively. Even at the smallest radii of 2.5, BaGoL begins to resolve the ring structure that is not visible in the traditional SR reconstructions. With the larger spacings, BaGoL resolves each emitter with a precision that improves with the number of localizations per emitter and with separation. Supplementary Fig. 5 shows the histogram of the number of found emitters obtained by analyzing 100 similar structures generated for each specific synthetic 8-mer. The reported precisions from BaGoL and the accuracies, as calculated by the deviation from the true position, improve with a number of localizations and spacing and are better than 1 nm for several conditions (Fig. 2 and Supplementary Fig. 6). The improvement with more localizations per emitter is related to both the ~1/sqrt(*λ*) factor and the reduced uncertainty in the number of emitters. The improvement with separation is related to both reductions in uncertainty of number of emitters and the reduction in uncertainty of the allocation of localizations to emitters. Moreover, the accuracy of the estimated emitter positions (difference between the true and estimated positions) are depicted in Supplementary Fig. 7, which again show improvement with increasing separations and the number of localizations per emitter.

In practice, it can be difficult to correct and entirely eliminate drift. Therefore, when nanometer emitter drifts are present in the data, our algorithm is capable of learning independent emitter drifts along with other parameters, which comes with the cost of an approximate factor of two loss of precision and accuracy (Supplementary Fig. 8). Supplementary Fig. 8i, j depicts the learned drifts by BaGoL in the *x* and *y* directions where peaks of the histograms deviate from the ground truth by <5%.

We explored the potential of BaGoL to improve resolution by improving precisions and resolve features of continuous structures with closely spaced emitters. Supplementary Fig. 9 shows the conventional SR and BaGoL results of simulated DNA-PAINT data of line pairs separated by two different distances, 6 and 12 nm, with average emitter separations on the lines of 1.4 nm. BaGoL clearly resolves the line pair separated by 6 nm whereas the conventional reconstruction does not.

For structures that are expected to be similar, BaGoL can be re-applied to aligned outputs from the MAPN where each MAPN emitter position is treated like a localization (Supplementary Figs. 10–11). Supplementary Fig. 10 illustrates this concept. Multiple individual MAPN results were aligned to a template (Supplementary Movie 2). The emitter positions and uncertainties returned from individual structures were treated like localization positions and uncertainties, and then grouped and combined using BaGoL to generate a high-precision estimate of the average structure. The resulting TUD structure matches that expected from the DNA-origami design^24^ with an average accuracy <1 nm. Supplementary Fig. 11 shows the results of this procedure applied to the DNA-rulers data set.

In Supplementary Fig. 13–14, we apply BaGoL to experimental dSTORM data from microtubules, the tetraspanin CD82 and labeled EGF bound to EGFR and use the resulting emitter positions to inspect the underlying structures and the spatial distribution of emitters. For the microtubule data, BaGoL reveals the parallel tracks structure expected from the 2D projection of a cylinder and that the sample is under-labeled (Supplementary Fig. 13a, b). From CD82 data, a region of the cell was selected for BaGoL analysis to investigate the spatial distribution, Supplementary Fig 13d, e. The BaGoL MAPN positions were used to calculate NND and the Hopkins’ statistic, both showing that CD82 is more regularly spaced than expected from a purely random (*i.e*. spatial Poisson) distribution (Supplementary Fig. 14a–f). Supplementary Fig. 13g, h shows BaGoL applied to dSTORM data of labeled EGF bound to EGFR. The small number of localizations per emitter does not allow BaGoL to dramatically improve precision, but the MAPN of BaGoL allows quantitative analysis. EGF induces dimerization in EGFR giving rise to an ~20 nm separation that includes the size of the EGF-streptavidin-biotin-Alexa647 label^27^. An inspection of the NND (Supplementary Fig. 14h) and Hopkins’ statistics (Supplementary Fig. 14i) of the MAPN coordinates shows a clustering behavior with an excess peak in NND at ~20 nm as compared to that expected from randomly distributed emitters (Supplementary Fig. 14b, c). Moreover, Supplementary Fig. 15 shows simulated dSTORM data of crossed lines with various linear labeling densities. Even with an average of just five blinking events before photobleaching, BaGoL improves upon conventional reconstruction. In addition, we compared BaGoL’s results with the results from the DDC algorithm^13^, which relies on photobleaching for enumerating emitters, using realistic simulated dSTORM data (Supplementary Fig. 16). In Supplementary Fig. 16b, DDC has modeled localizations further away from the cross (ground truth locations) as single emitters likely due to the lack of uncertainty propagation from localization precisions in the DDC algorithm. We also quantitatively assessed the performances by calculating the JAC and RMSE for both algorithms. The resulting JACs are, respectively, 0.85 and 0.80 for BaGoL and DDC. Further, the resulting RMSEs are, respectively, 12.9 and 13.2 nm for BaGoL and DDC. This implies that BaGoL slightly outperforms DDC for dSTORM data but they are still comparable.

The performance of the NND filter used to remove outliers is illustrated in Supplementary Fig. 17. Supplementary Fig. 17a, b depicts localizations from the MPI structure in Fig. 2j, and the histogram of the number of localizations within three median of localization precisions. The red dots in Supplementary Fig. 17a are localizations that fall to the left of the valley in the histogram, indicated by the red arrow, and are recognized as outliers. Supplementary Fig. 17c, d, respectively, show the posterior image of BaGoL before and after applying the NND outlier removal filter. Comparison of panels c-d demonstrates worse precisions and spurious emitters in the absence of the filter. Supplementary Fig. 17e–h depicts the same set of results for the TUD structure shown in Supplementary Fig. 10. In this case, the resulting posterior image without applying BaGoL also shows excess emitters and worse precisions in the presence of outliers in Supplementary Fig. 17g.

## Discussion

Grouping localizations to improve precision is a simple concept, but in practice requires the proper treatment of uncertainties in both the number of true emitters and the allocation of localizations to emitters. There are many clustering algorithms that could be employed for this problem such as hierarchical, k-means, Gaussian mixture models^28^, DBSCAN^29^, Voronoi tessellation^30^, and others. None of these general purpose clustering algorithms make best use of the information available in SMLM data, particularly the variable localization precision and information about the distribution of localizations from an emitter. We tested several of these algorithms and did not obtain satisfactory results (Supplementary Fig. 12). Therefore, we employed the RJMCMC approach within the Bayesian paradigm described here, which allows propagation of uncertainties, e.g., uncertainties in allocations of localizations to emitters, throughout the problem and does not require the number of emitters a priori.

The core RJMCMC step of the BaGoL algorithm makes the assumption that localizations are generated by the underlying true emitter, the emitter is labeled by a single dye or docking strand, and that the localization uncertainty is reported correctly. We chose to primarily use DNA-PAINT to demonstrate the quantitative aspect of BaGoL experimentally because it is possible to label proteins with only one docking strand and the binding kinetics are independent of laser intensity (such as might vary across the image or with depth in total internal reflection microscopy) and of buffer conditions such as oxygen or thiol concentration. We also expect that the number of binding events per docking strand will be well described by a Poisson distribution^31^, although any distribution can be used with BaGoL. Experimental SMLM data can provide additional challenges due to spurious SMLM localizations that do not correctly originate from a static, true emitter. Particularly detrimental are high-precision but inaccurate localizations that can arise from fitting two emitters in the raw data as a single emitter. Here, we used preprocessing to identify and remove probable double-fitting events. In practice, it would be desirable to image at a low enough duty cycle so that these events are rare and/or use of a multiple emitter fitting method that could identify these events as multiple emitters. Moreover, small, nanometer scale movements of individual emitters are present for many emitters in some of the data sets and required modeling these movements to avoid mis-representation by an excess of emitters. Resolving these issues allowed BaGoL results of experimental data to approach that of synthetic data.

The grouping of localizations into emitters makes it possible for downstream analysis such as cluster analysis of the resulting emitter positions (Supplementary Figs. 13–14). BaGoL is particularly suited for the quantitative analysis of small oligomers, such as dimers, separated by several nanometers (Supplementary Fig. 1). In principle, imaging longer would generate more localizations leading to higher precision. In practice, the precision seems to be limited by sample fixation and nanoscale movements. As a rule of thumb, we would recommend targeting about 50 localizations per emitter with an anticipation of ~1 nm precision.

In this work, BaGoL was applied to 2D data. However, the algorithm can be extended in a simple manner to any number of dimensions, most obviously to include the axial direction. This could provide nanometer precision in the axial dimension comparable to that from interference-based measurements^32^. Applications to other dimensions can also be envisioned, such as overlapping spectral data. The sub-nanometer precision of BaGoL could be combined with other experimental approaches to generate independence and sparsity on the scale of nanometer precision, such as multicolor imaging or sequential imaging using orthogonal docking strands. It is also possible to use localizations’ timestamps in our likelihood model, but for imaging modalities with no photobleaching, e.g., DNA-PAINT, this will not yield any further benefits and may only provide minimal benefit with dSTORM as evidenced by our comparable performance with the DCC algorithm (Supplementary Fig. 16). Although grouping of localizations in PALM data could also yield benefits^14^, and the BaGoL algorithm could be applied to SMLM data from PALM, we did not explore BaGoL performance with real or synthetic PALM data in this manuscript.

Finally, BaGoL performance scales with the available information in the input data sets. This formalism can make direct use of future improvements in localisation precision and labeling accuracy, density and stoichiometry.

## Methods

### BaGoL implementation

BaGoL was implemented in MATLAB (MathWorks Inc.) using object oriented programming. The stages of the algorithm were organized as methods and functions of a class. All the methods, with the exception of the frame connection algorithm, were implemented as MATLAB m-files and require the MATLAB Statistics and Machine Learning toolbox. The frame connection algorithm was written in C++ and was compiled into a mex-file that could be used within MATLAB. A desktop computer with an i7, 3.64 GHz CPU was used to process both simulated and experimental data. The software is included as Supplementary Software 1. The algorithm took ~8 min to analyze the MPI DNA-origami structure in Fig. 2.

### BaGoL analysis

The probabilities for proposing different jump types were (*P*_Move_, *P*_Allocate_, *P*_Birth_, *P*_Death_)=(0.25, 0.25, 0.25, 0.25). The localizations per emitter distribution were either deduced simultaneously along with the other parameters, or learned from a part of the data itself and then used to analyze the entire data set by BaGoL. This is specific for each data set as described in their individual sections. For experimental DNA-PAINT data, we removed outlier localizations using the NND filter. Outliers were identified based on the number of localizations within three times the median of the localization precisions. This procedure is as follows: (1) calculate the number of localizations within three times the median of the precisions, denoted by *Ψ*, for every localization; (2) find the valley in the histogram of the set of obtained *Ψ*; (3) use the valley as a starting point to find a threshold and eliminate localizations that have less neighbors than the threshold. This is illustrated in Supplementary Fig. 17. For some data sets, this procedure results in a little larger threshold value than the valley within the histogram of *Ψ* allowing a more aggressive outlier removal. The NND filter was not used for dSTORM data due to the low number of blinking events per emitter. Furthermore, to generate synthetic data, we used a PSF size of 120 nm and 1800 photons per blinking/binding event throughout this work except where mentioned otherwise.

### DNA origami

DNA-origami data was collected using μManager^33^. All DNA-origami data except MPI structures were localized using the BAMF algorithm^23^. The MPI data was localized using PICASSO^7^. DNA-origami structures of the TUD pattern (Fig. 2g) and grid pattern (Fig. 2d) were part of the same data set, so we used the grid to estimate the distribution of localizations per emitter, *ξ*. We did so by manually selecting multiple isolated grids and the average number of localizations per emitter were found by dividing the total number of localizations by the number of docking strands in those grids. The resulting mean value, *λ* ~ 85, was used to parameterize a Poisson distribution prior for the localization per emitter distribution. This data set was originally collected for our previous work^24^ and we removed outliers using the same procedure as described in this paper. The isolated grids and TUD structures were then manually picked and processed by BaGoL. The localization precisions were inflated by 1.25 nm to compensate for what appeared to be an under-reported localization precision. To analyze the small and large grids, we took 1000 samples for both burn-in and post burn-in chains. For TUD structures, 5000 samples for each burn-in and post burn-in chain were taken from the posterior. The MAPN coordinates from BaGoL for 170 TUD structures were aligned with a template generated from origami design (Supplementary Fig. 10). The collection of aligned coordinates and their associated uncertainties were again processed by BaGoL using the mean number of binding events *λ* = 150, which is approximately the number of aligned TUD patterns. The precisions were inflated by 0.8 nm, while this time the outliers were removed using the NND filter as described in Supplementary Fig. 17.

For the MPI structure (Fig. 2j), the distribution of localizations per emitter (*ξ*) was simultaneously estimated along with the other parameters. The outliers were removed using the NND filter, as described in Supplementary Fig. 17. The precisions were adjusted by inflating them by 0.4 nm.

### DNA rulers

The DNA-ruler data was collected and localized using custom-written software packages MIC^34^ and SMITE^35^. 150 isolated DNA-rulers were picked manually and analyzed by BaGoL. The distribution of localizations per emitter was fixed by adjusting the mean number of binding events *λ* = 50, obtained the same way as explained in the previous section. Outliers were removed using the NND filter, localization precisions were increased by 2.5 nm and no docking strands drifts were permitted. An example of raw data and BaGoL results are presented in Fig. 2a–c. The MAPN coordinates from the DNA rulers were then shifted and rotated to match a template with the known spacing, Supplementary Fig. 11. Not all DNA rulers in the test sample were formed correctly. The DNA rulers that did not match the template, defined as structures where the sum of their nearest neighbor distances with the template were more than 6 nm, were removed from the set of aligned DNA rulers. The collection of the MAPN coordinates from the aligned structures was then analyzed by BaGoL using *λ* = 50.

### DNA-PAINT data analysis of kindlin

A 5 × 5 μm^2^ region of data was selected. Localizations were frame-connected^36^ and those not connected to anything were removed. The localizations were further filtered by the NND filter. We inflated the precisions by 2.5 nm to compensate for under-reported precisions. The data was analyzed using a subregion size of 500 nm and taking 10,000 samples from the posterior for each sub-region. The hierarchical Bayes method, Supplementary Note 2.2, was used to learn the localization per emitter distribution.

### Jaccard index and root mean square error

To obtain the JAC and RMSE plots in Fig. 4, data sets with 9 logarithmically spaced concentrations, starting from 1000 emitters per μm^2^ to 17,000 emitters per μm^2^, were generated (average nearest neighbor separations can be found as <r > = 1/(2sqrt(*ρ*)), where *ρ* represents the concentration with respect to area). An example of the synthesized data is presented in Fig. 4b. For every concentration, 5 data sets were synthesized using average number of blinking events *λ* = 15, 50 per emitter, over an area of 500 × 500 nm^2^. Data sets were analyzed by BaGoL using subregion sizes of 50 × 50 to 20 × 20 nm^2^, for concentrations ranging from 1000 emitters per μm^2^ incremented logarithmically to 17,000 emitters per μm^2^, and taking 30,000 samples from the posterior. The hierarchical Bayes method was used to learn the localization per emitter distribution. JAC and RMSE for each data set were calculated using pairs of matched emitters from the ground truth set and the set of MAPN emitters, where the matched emitters were identified by minimizing the distance cost matrix between the true and MAPN emitters using the Hungarian algorithm^37^ and pairs with costs no more than three times the average of localization precisions returned by BaGoL.

### Generation and analysis of synthetic dimers

Two groups of localizations with *λ* = 50, separations of 1, 2, 5, and 10 nm were simulated, Supplementary Fig. 1. The number of localizations per emitter and the average intensities of the localizations were drawn from a Poisson and an exponential distribution, respectively, with the given means. The produced data sets were then processed using a fixed distribution of localizations per emitter by setting the mean of *ξ* to the given *λ* values and taking 20,000 samples from the posterior.

### Generation and analysis of synthetic 8-mers

Synthetic 8-mers with radii of 2.5, 5, 10, and 20 nm were generated to mimic positions and uncertainties that would result from a standard SMLM experiment (the distance between neighboring emitters in an 8-mer is ~0.76 *R*, where *R* is the radius). The number of binding events from each emitter was drawn from a Poisson distribution parameterized by the expected number of events per emitter, *λ* = 10, 20, and 50. For each binding event, the number of photons collected, *I*, was drawn from an exponential distribution with an average intensity of 1800 photons. The localization precision of the blinking/binding event for each dimension was calculated using σ = σ_PSF_/sqrt(*I*). The observed location of the blinking/binding events were generated by drawing a value from *N*(*y*,σ^2^) where *y* is the true position at the time of the event and *N* is the normal distribution. These data sets were employed to make Supplementary Figs. 2–7. The produced data sets were processed using hierarchical Bayes to learn the localization per emitter distributions.

The depicted accuracies in Supplementary Fig. 7 are the distances of the found MAPN positions from the true positions. These data are compared to the predicted distribution f(r) = r/σ^2^ exp(-(r^2^/2σ^2^)) (magenta curves), in which the parameter σ was taken as the precision mean from the corresponding simulations in Supplementary Fig. 6. The distributions were scaled to have the same areas under the curve as the accuracy data over the data ranges displayed.

### Double crossed lines

The simulated cross was composed of double lines with separations of 6 and 12 nm and lengths of 100 nm, Supplementary Fig. 9. Seventy emitters were placed at random positions along each line, producing an average nearest neighbor distance of ~1.4 nm. The emitters were synthesized with *λ* = 50 average number of localizations per emitter. The generated data set was then processed with a Poisson prior on the number of localizations per emitter with a fixed mean *λ* = 50, taking 30,000 samples from the posterior.

### Structure alignment

Structure alignment was performed by minimizing the nearest neighbor distances between a template structure and the experimentally obtained structures. An experimental structure was first moved so that its center of mass matched with the center of mass of the template. It was then iteratively rotated and translated by random amounts to minimize the sum of the nearest neighbor distances between the structure and the template vertices using a Monte Carlo approach. The contributions of localizations to the sum with nearest neighbors further than a cutoff distance were set to the cutoff distance. The length of the chain was 3000 samples. For the first and second half of the chain, we, respectively, used rotation and translation jump sizes of 1 and 0.1 radian, 0.5 and 0.05 nm. The second half of the chain was then employed to calculate the rotation and translation that minimize the sum of nearest neighbor distances. Examples of alignment of TUD-structures and DNA-rulers are, respectively, presented in Supplementary Figs. 10–11.

### Comparison of clustering algorithms

A synthetic 8-mer was generated as described above with a radius of 10 nm and *λ* = 50. This data set was analyzed using five different algorithms as shown in Supplementary Fig. 12. The data set was processed with BaGoL using the parameters *λ* = 50 and taking 10,000 samples from the posterior. For the algorithm described in Rubin-Delanchy et al.^38^, we used the recommended value, 20, for the Dirichlet prior and a gamma distribution with a mean of 4 nm, which was the average of the localization precisions. For DBSCAN, the mean number of data points within a group was set to (*λ*−2sqrt(*λ*))/2 and the distance parameter *ɛ* was adjusted to be the average of the localization precisions. The best result from 10 different runs of *k*-means with 150 iterations and 8 groups is depicted in Supplementary Fig. 12e. The Gaussian mixture model algorithm was run with 8 groups.

### dSTORM data of biological samples

All the dSTORM data was collected using MIC^34^ and localized using SMITE^35^ (custom-written software packages). Localizations with intensities more than 3000, 2000 and 4000 photons were removed from the lists of coordinates, respectively, for microtubules, CD82 and EGFR, Supplementary Fig. 13. For these data sets, we first ran BaGoL to learn *ξ*, numbers of blinking events per emitter distribution, via the hierarchical Bayes scheme using less dense regions of the same data sets. The returned *ξ* values were then used in second runs of BaGoL where each data was processed by taking 5000 samples from the posterior. The localization precisions were increased by 1, 2 and 0.75 nm, respectively, for microtubule, CD82 and EGFR data.

We also inspected the cluster formation and protein interaction in CD82 and EGFR data using the MAPN coordinates of emitters from BaGoL; Supplementary Fig. 14. We calculated the nearest neighbor distribution for both sets of MAPN coordinates, using the built-in MATLAB function *knnsearch*, and compared them with uniform randomly distributed data, Supplementary Fig. 14b, e, h. Hopkins’ statistics^39^ were also utilized to examine clustering in biological samples. The Hopkins’ statistic (*H*) tests for spatial randomness of a point pattern by comparing nearest neighbor distances from random emitters and randomly chosen positions. Supplementary Fig. 14c, f, i shows the PDF of *H* for 1,000 iterations of random emitters and location choices (blue) of simulated data compared to the analytic curve (red) for pure random data. Values of *H* near 0.5 imply randomly distributed data, while values near 1 indicate highly clustered data, and values near zero signify more regularly spaced data.

### dSTORM data of cross in the presence of bleaching

dSTORM data was simulated considering an on-state, an off-state and a bleached-state for every fluorophore with off to on rate *K*_on_ = 2 × 10^−4^, on to off rate *K*_off_ = 5 × 10^−2^, and on to bleach rate *K*_b_ = 0.05, respectively, Supplementary Fig. 15. The life-time of the on-state and off-state were sampled from exponential distributions with the given rates. A fluorophore in on-state can transit to either the bleached-state or off-state. Therefore, we used the Gillespie algorithm^40^ to simulate the transition from the on-state to the next state using the rates *K*_off_ and *K*_b_. The data were simulated over 100,000 frames so that the entire set of fluorophores were bleached. The fluorophores were equally spaced on the cross with three different separations of 5 nm, 10 nm and 15 nm. The resulting number of blinking events per fluorophore have exponential distributions with means of ~5. We used the same exponential distribution as the prior on number of blinking events per emitter by taking a gamma prior with η = 1 and γ = 5, see Supplementary Note 1 for a description of the parameters.

### Experimental data collection

#### DNA rulers

GATTA-PAINT nanoruler slide samples (HiRes 20R, GattaQuant DNA Technologies) were used as purchased. Imaging was done on an Olympus IX71 inverted wide field fluorescence microscope setup as described previously^41^. Fluorescence excitation of the sample was done using a 642 nm laser diode (HL6366DG, Thorlabs). The laser beam was collimated and passed through a multi-mode fiber (P1-488PM-FC-2, Thorlabs), before being focused on the back focal plane of a 1.45 NA oil objective (UAPON 150XOTIRF, Olympus America Inc.). TIRF excitation of the sample was achieved by translating the laser close to the edge of the objective back aperture. Fluorescence emission collected from the nanoruler sample was passed through a quad band dichroic/emission filter set (LF405/488/561/635-A; Semrock, Rochester, NY) and a band pass filter (685/45, Brightline) before being detected using an EM CCD camera (iXon 897, Andor Technologies). A total of 100,000, 256 × 256 pixel frames were collected using a 100 ms exposure time. Data collection on the microscope was controlled by custom-written MATLAB instrument control software (MIC)^34^. The raw super-resolution data of DNA rulers was processed by a single-emitter fitting algorithm^42^ and thresholded by *p*-value and localization uncertainty^43^. Localizations from the same binding events were combined using a frame connection algorithm.

#### DNA-origami

DNA-PAINT data was collected as described in refs. 24, 25. The raw super-resolution data for the origami grids and TUD logos were analyzed using the BAMF multiple-emitter fitting algorithm^23,24^. The raw super-resolution data of MPI logos were analyzed using the PICASSO package^7^. The resulting localizations were then combined across consecutive frames using a frame connection algorithm. Localizations that were not connected across at least two frames were filtered out.

#### DNA-PAINT kindlin-2-GFP

Cells were maintained in high glucose Dulbecco’s modified Eagle’ medium (Thermo Fisher, 31966047) supplemented with 10% fetal bovine serum (Thermo Fisher, 10270106) and 1% penicillin/streptomycin (Sigma, P4333). Kindlin-2-GFP constructs were stably expressed together with talin-1-RFP by retroviral infection in quadruple knockout fibroblasts deficient for talin-1, talin-2, kindlin-1, and kindlin-2(Tln1 − /−Tln2−/−K1−/− K2−/−)^44^. Kindlin-2-GFP was labeled for DNA-PAINT imaging by a GFP nanobody conjugated to the R1 DNA-PAINT sequence to label the cells as described in ref. 25. For imaging, 40,000 cells were seeded on ibidi 8-well glass bottom slides (ibidi, 80807) as described in ref. 26. 80,000 frames were recorded at 100 ms integration time using 200 pM R1-Cy3b imager. Data was acquired on an inverted Nikon Eclipse Ti microscope (Nikon Instruments) with the Perfect Focus System, operated with an objective-type TIRF configuration with an oil-immersion objective (CFI Apo TIRF 100×/1.49-NA). Samples were excited with a 561-nm laser (Coherent Sapphire). The laser beam was passed through a cleanup filter (ZET561/10; Chroma Technology) and coupled into the microscope objective with a beam splitter (ZT561rdc; Chroma Technology). Fluorescence light was spectrally filtered with two emission filters (ET600/50 m and ET575lp; Chroma Technology) and imaged on an sCMOS (scientific complementary metal-oxide semiconductor) camera (Zyla 4.2plus; Andor Technologies). Imaging was performed without additional magnification in the detection path and 2 × 2 camera binning, resulting in a pixel size of 130 nm.

##### dSTORM microtubules

HeLa cells were plated on a #1.5 coverslip in growth media and were incubated overnight. The cells were washed once with PBS and then fixed by a two-step fixation: 60 seconds in a solution of 0.6% paraformaldehyde, 0.1% glutaraldehyde, 0.25% Triton X-100 in PBS, followed by 1.5 h in a solution of 4% paraformaldehyde and 0.2% glutaraldehyde in PBS. The cells were then washed twice with PBS followed by 5 min in a solution of 0.1% NaBH4 in PBS. The cells were again washed twice with PBS followed by two 5 min washes in a solution of 10 mM Tris in PBS. The cells were then washed twice with PBS followed by a 15 min wash in a solution of 5% BSA and 0.05% Triton X-100 in PBS. The cells were washed once with PBS and then labeled for 1 hour in a solution of 2% BSA, 0.05% Triton X-100, and 2.5 μg/mL of anti-α tubulin-Alexa647 (NOVUS Biologicals, NB100-690AF647) in PBS. The cells were then washed three times for 5 min each in a solution of 2% BSA and 0.05% Triton X-100 in PBS. Imaging was performed in a standard dSTORM imaging buffer with an enzymatic oxygen scavenging system and primary thiol: 50 mM Tris, 10 mM NaCl, 10% w/v glucose, 168.8 U/ml glucose oxidase (Sigma #G2133), 1404 U/ml catalase (Sigma #C9322), and 60 mM 2-aminoethanethiol (MEA), pH 8.5. Data was collected as described for the DNA-rulers with 16 ms exposure time and a total of 200,000 frames. Weak 405 nm light was used to accelerate emitters out of the dark state.

##### dSTORM CD82

HEK 293 cells stably overexpressing mCherry-CD82^45^ and EGFR-EGFP^46^ were plated on fibronectin coated eight-well Lab-Tek chamber slides overnight at 37 °C. Cells were fixed with 4% PFA, blocked with 3% BSA/PBS and labeled with Alexa Fluor 647 anti–human CD82 antibody (BioLegend, ASL-24) at 1.0 μg/mL. Cells were washed and fixed again with 4% PFA. Labeled cells were imaged in a reducing buffer composed of 50 mM Tris, 10 mM NaCl, 10% w/v glucose, 168.8 u/ml glucose oxidase (Sigma #G2133), 1404.0 U/ml catalase (Sigma #C9332), and 20 mM MEA, pH 8.5. Cells were imaged using a custom TIRF microscope system as described for the DNA-rulers. 40,000 frames were collected per cell, with a brightfield image acquired every 2000 frames for registration and drift correction. Each frame was 256  × 256 pixels with a pixel size of 0.107 µm and an acquisition time of 16 ms per frame.

##### dSTORM EGF

CHO cells stably expressing EGFR-GFP^46,47^ were plated overnight on piranha cleaned 25 mm coverslips. Cells were washed with PBS and then treated with 50 nM AlexaFluor647-conjugated EGF (ThermoFisher Scientific, #E35351) for 8 min at room temperature to ensure dimerization of EGFR on the basal surface of the cell^11^. Cells were washed with PBS and immediately fixed with 4% paraformaldehyde for 2 h. Finally, cells were washed two times with PBS and once with 10 mM Tris-HCl (pH 7.2) and stored in PBS until imaging. Imaging was performed in a standard dSTORM imaging buffer with an enzymatic oxygen scavenging system and primary thiol: 50 mM Tris, 10 mM NaCl, 10% w/v glucose, 168.8 U/ml glucose oxidase (Sigma #G2133), 1404 U/ml catalase (Sigma #C9322), and 60 mM 2-aminoethanethiol (MEA), pH 8.5. Data was collected using a custom-built microscope consisting of a 647 nm laser excitation source (500 mW 2RU-VFL-P; MPB Communications Inc.), an sCMOS camera (C11440-22CU; Hamamatsu Photonics) a 1.35 NA silicon oil immersion objective (Olympus UPLSAPO100XS) and a 708/75-nm emission filter (FF01-708/75-25; Semrock). A total of 60,000 frames were collected with an exposure time of 50 ms per frame.

### Reporting summary

Further information on research design is available in the Nature Portfolio Reporting Summary linked to this article.

## Supplementary information

Supplementary Information

Description of Additional Supplementary Files

Supplementary Movie 1

Supplementary Movie 2

Supplementary Software 1

Reporting Summary

## Data Availability

The data that support this study are available from the corresponding authors upon reasonable request. Source data are provided with this paper.

## Source data

Source Data

## Code availability

The BaGoL software package is included as Supplementary Software 1 and also available on Github (https://github.com/LidkeLab/BaGoL). Some of the data sets were collected and localized using custom-written software packages of MIC and SMITE, available on Github (MIC: https://github.com/LidkeLab/matlab-instrument-control) and (SMITE: https://github.com/LidkeLab/smite).
