# Supplementary material for: High-precision estimation of emitter positions using Bayesian grouping of localizations
Source: Nat Commun. 2022 Nov 22;13:7152. doi: 10.1038/s41467-022-34894-2 (PMC9684143; doi:10.1038/s41467-022-34894-2)
Supplement: Supplementary file 1 — Supplementary Information [file 41467_2022_34894_MOESM1_ESM.pdf]

Supplementary Information
High-Precision Estimation of Emitter Positions using Bayesian Grouping of Localizations

Mohamadreza Fazel[1], Michael J. Wester[1,2], David J. Schodt[1], Sebastian Restrepo Cruz[3], Sebastian Strauss[4,5], Florian Schueder[4,5], Thomas Schlichthaerle[4,5], Jennifer M. Gillette[3,6], Diane S. Lidke[3,6], Bernd Rieger[7], Ralf Jungmann[4,5], and Keith A. Lidke[1,6*]

[1] Department of Physics and Astronomy, University of New Mexico, Albuquerque, New Mexico, USA.
[2]Department of Mathematics and Statistics, University of New Mexico, Albuquerque, New Mexico, USA.
[3]Department of Pathology, University of New Mexico Health Science Center, Albuquerque, New Mexico, USA.
[4]Department of Physics and Center for Nanoscience, Ludwig Maximilian University, Munich, Germany.
[5]Max Planck Institute of Biochemistry, Martinsried, Germany.
[6]Comprehensive Cancer Center, University of New Mexico, Albuquerque, New Mexico, USA.
[7]Department of Imaging Physics, Delft University of Technology, Delft, the Netherlands.

November 2, 2022

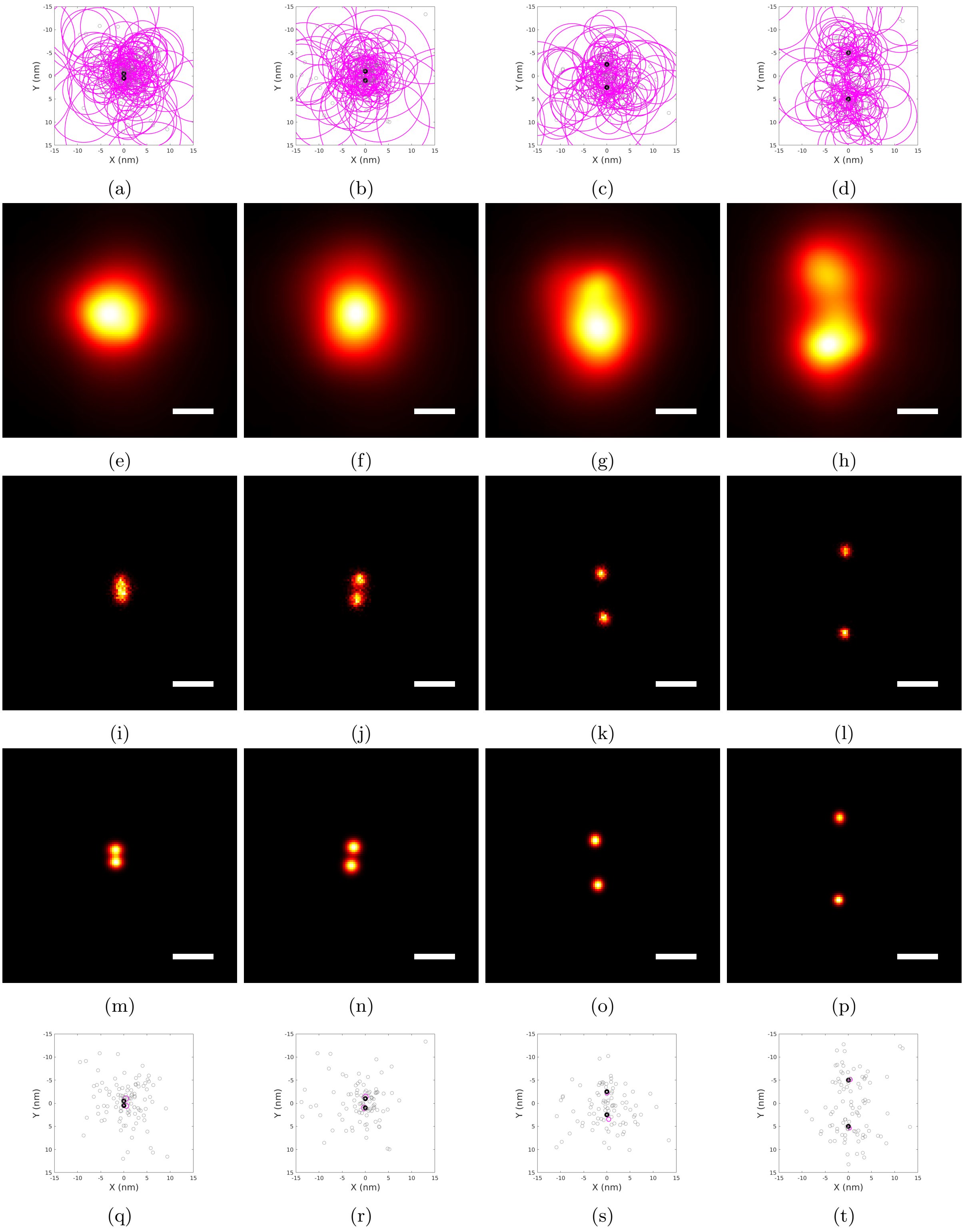

Supplementary Figure 1: BaGoL applied to two closely spaced emitters (two-point resolution). Two emitters are simulated at 1, 2, 5 and 10 nm separations (left to right columns) with an average $\lambda = 50$ localizations per emitter and 1800 photons per localization. In all plots true emitter positions are shown as black circles and localizations are shown as gray circles. Magenta circles represent a 1 σ localization precision. (a-d) Observed localizations. (e-h) Traditional super-resolution images. (i-l) BaGoL Posterior Images. (m-p) BaGoL MAPN images. (q-t) Plots of MAPN results from BaGoL. BaGoL analysis was repeated for $N = 5$ similar structures. Scale bars are 5 nm.

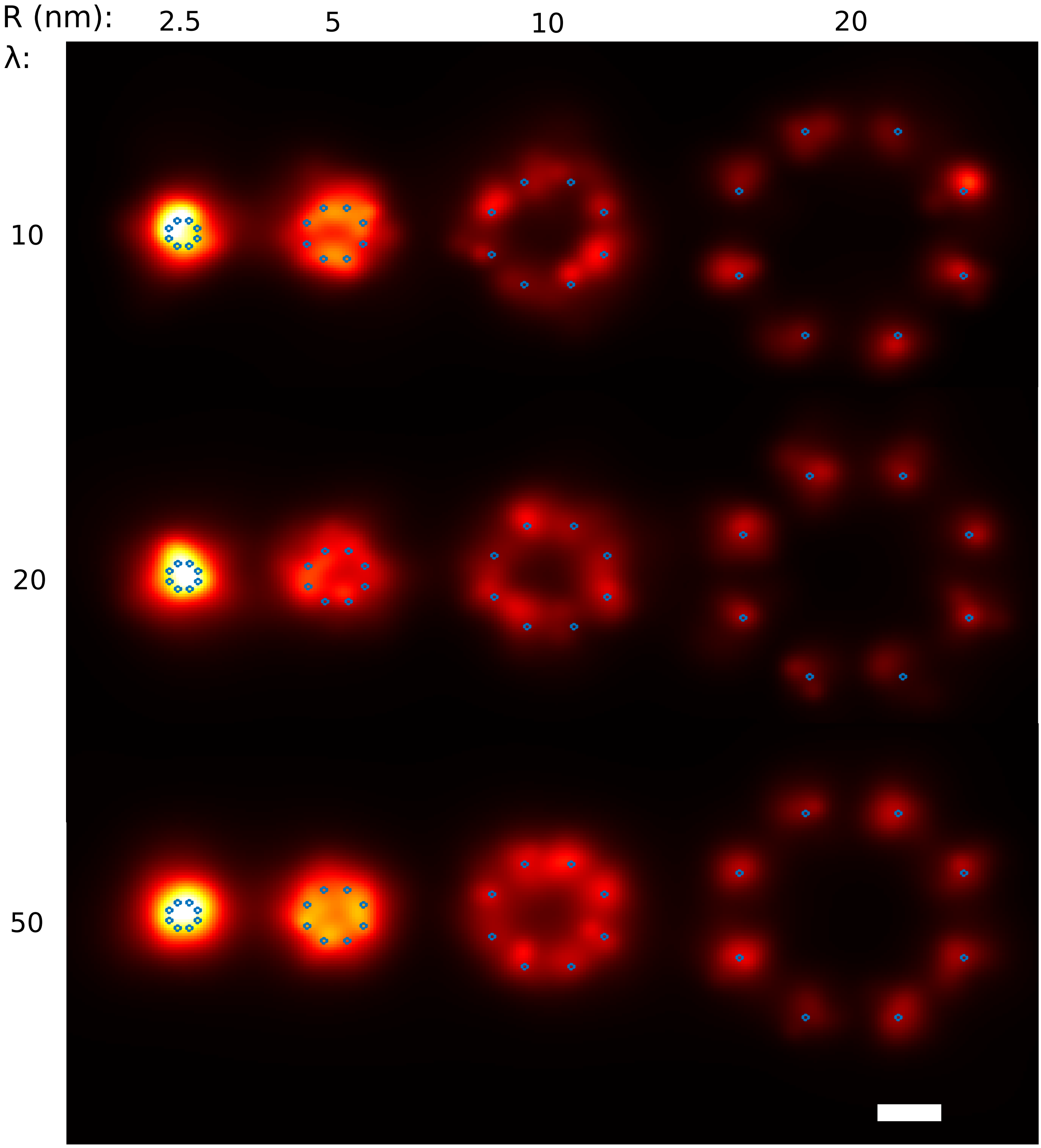

Supplementary Figure 2: Traditional super-resolution reconstructions of simulated 8-mers. The number of blinking/binding events for each row are drawn from a Poisson distribution by the same mean λ, and columns have the same radii R. The nearest neighboring emitter distance is given by $\sim 0.76R$. The blue circles show true emitter positions. The average number of photons (intensity) per blinking/binding event and the PSF size are, respectively, 1800 photons and 120 nm. The 8-mers on each row were analyzed simultaneously using BaGoL to benchmark its performance in Supplementary Figs. 3-7. The same parameters and convention are used in Supplementary Figs. 2-7. BaGoL analysis was repeated for $N = 100$ similar structures. The scale bar is 10 nm.

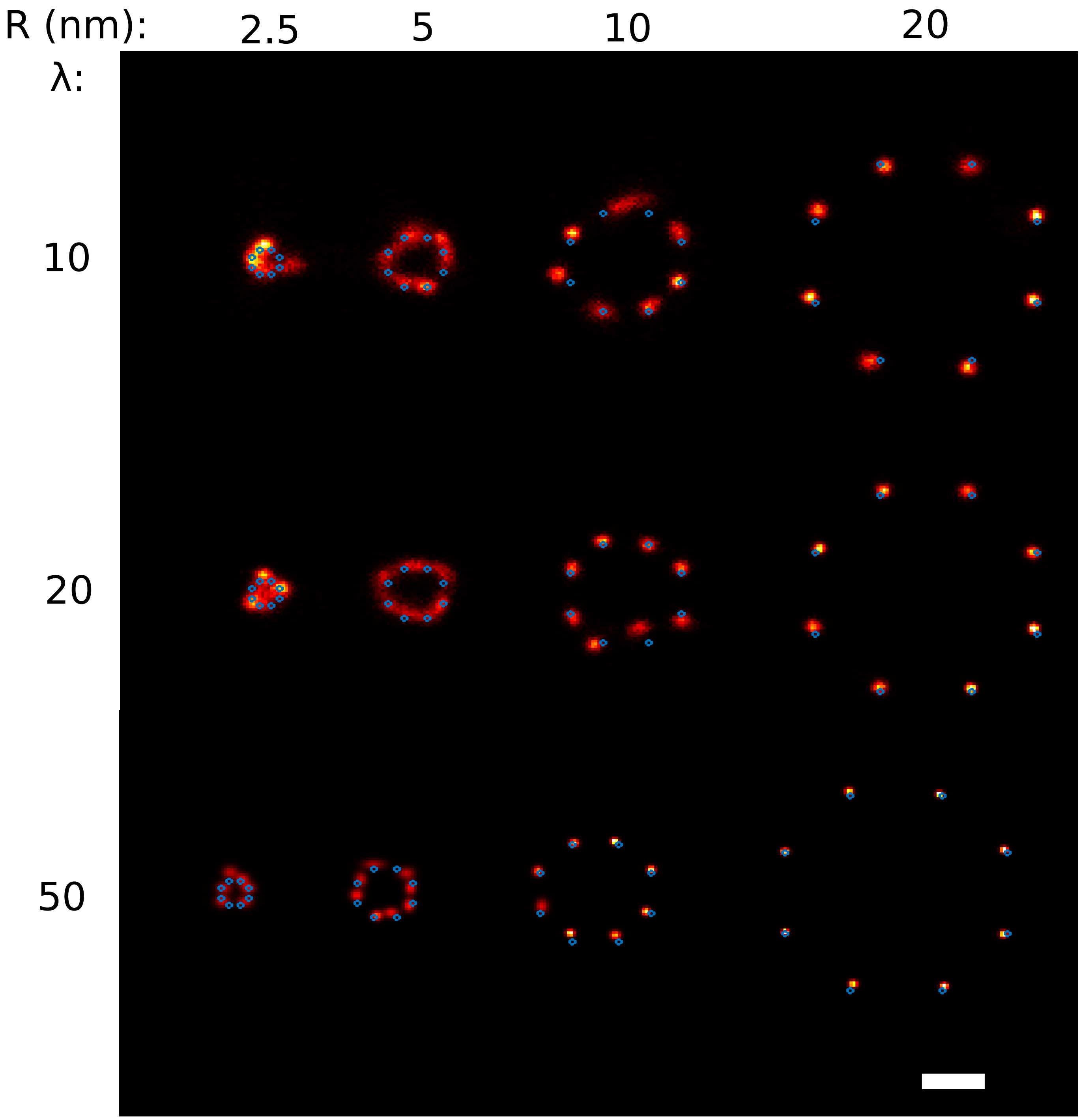

Supplementary Figure 3: BaGoL posterior images corresponding to the same data shown in Supplementary Fig. 2.

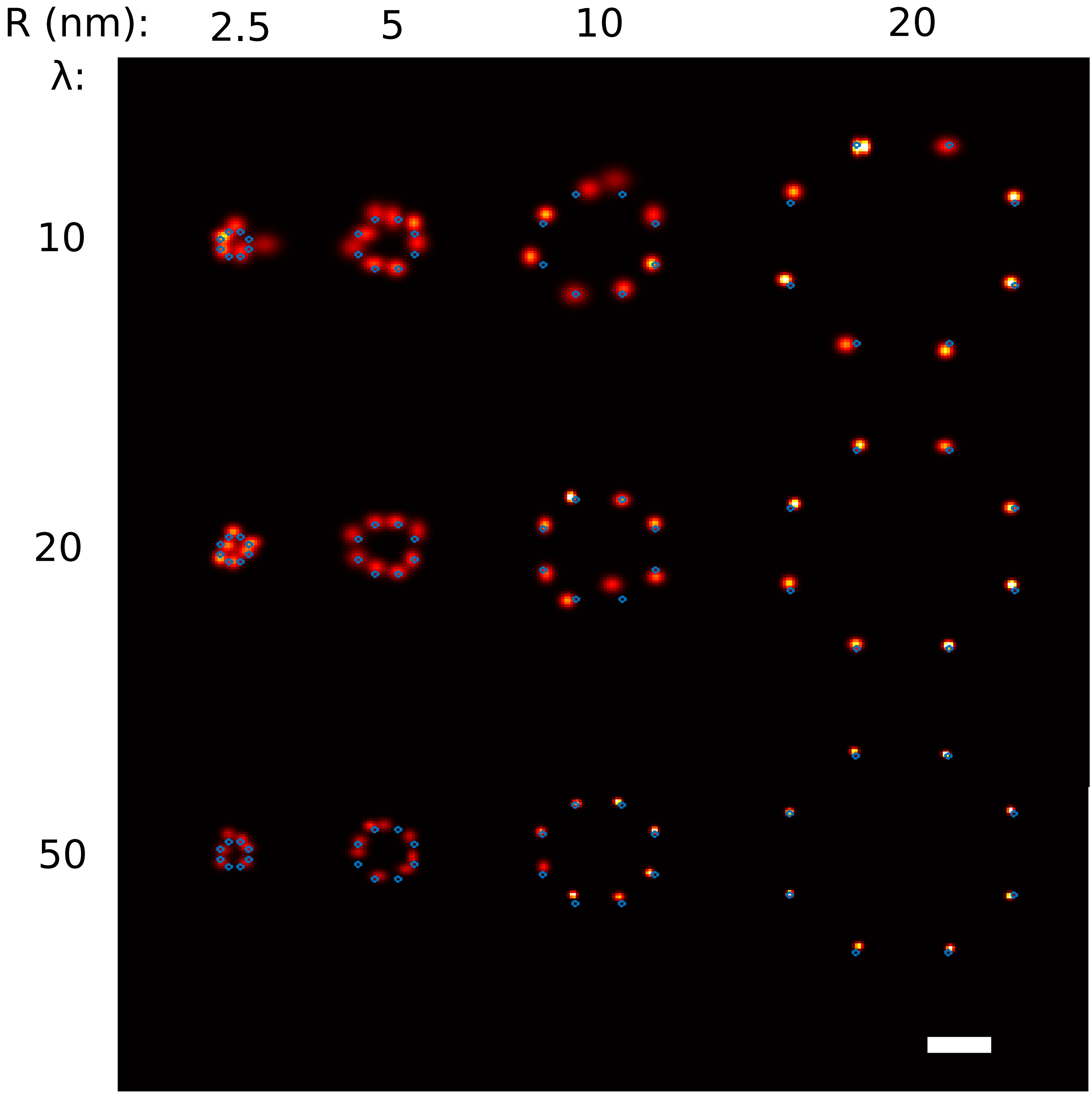

Supplementary Figure 4: BaGoL MAPN images corresponding to the same data shown in Supplementary Fig. 2.

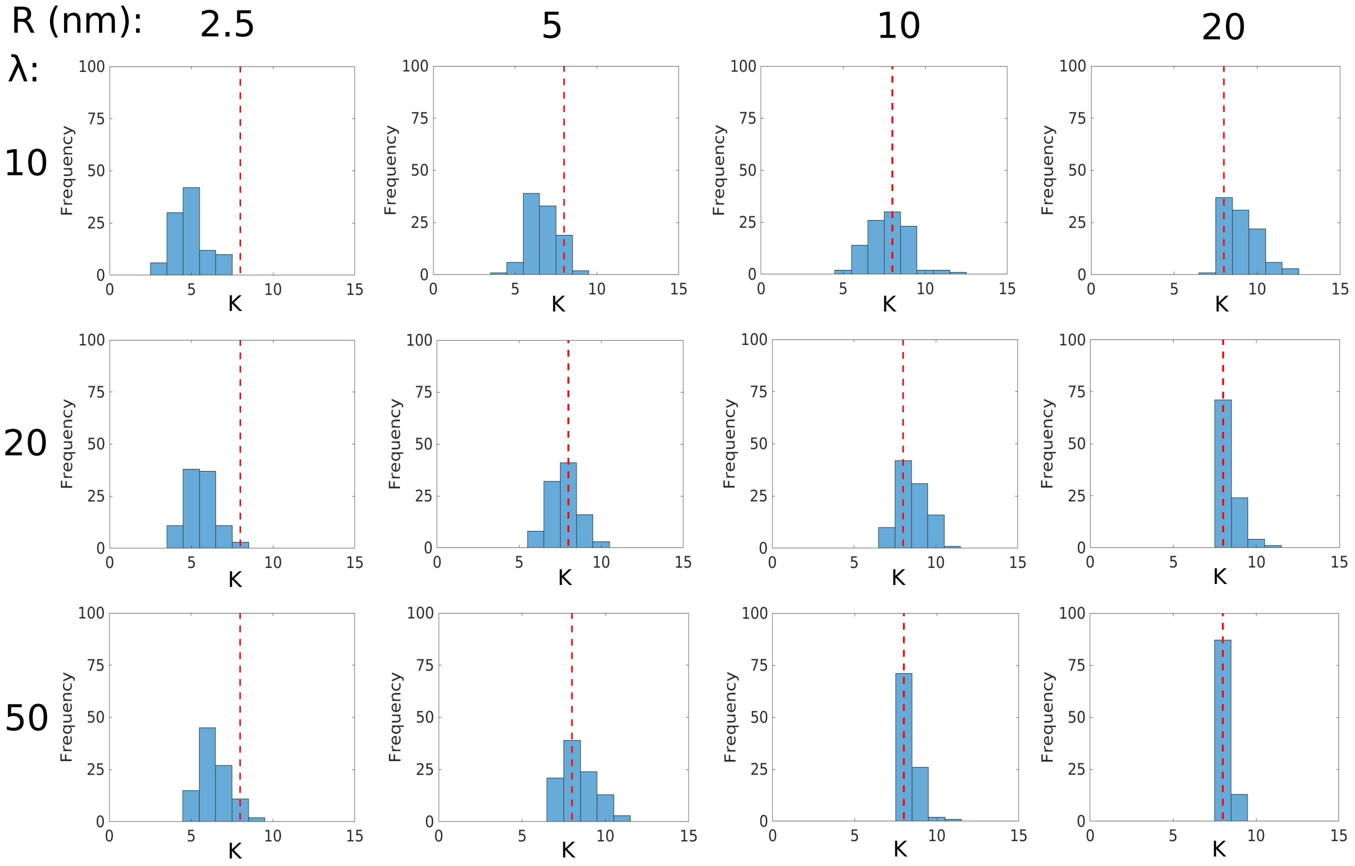

Supplementary Figure 5: Histogram of the number of found emitters for $N = 100$ 8-mer structures similar to the MAPN images in Supplementary Fig. 4. K denotes the number of found emitters. The red dashed lines represent true number of emitters.

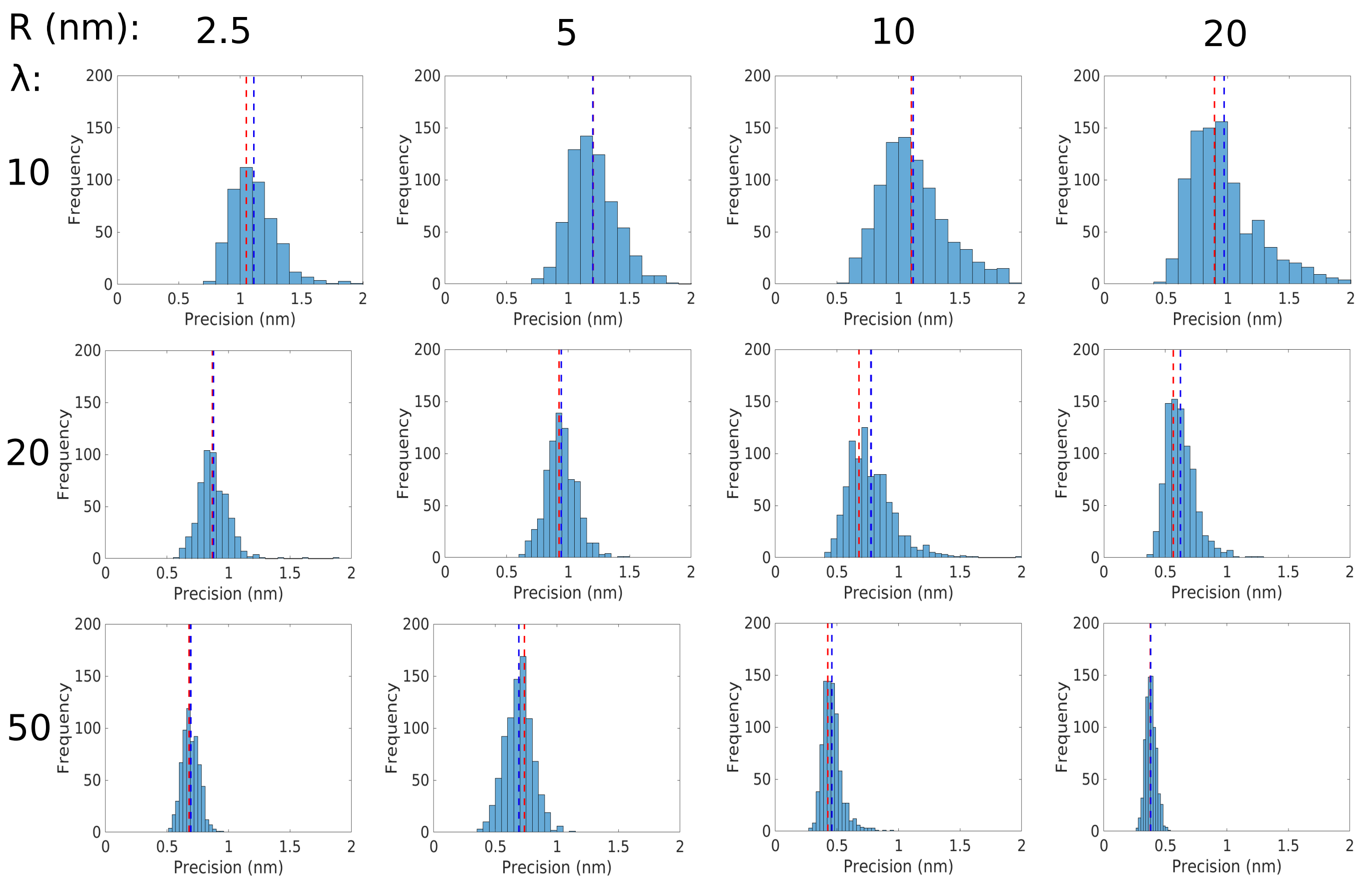

Supplementary Figure 6: Histogram of precisions for $N = 100$ 8-mer structures similar to the MAPN images in Supplementary Fig. 4. The red and blue dashed lines, respectively, show the mode and the mean of the histograms. The same convention is followed in Supplementary Fig. 7.

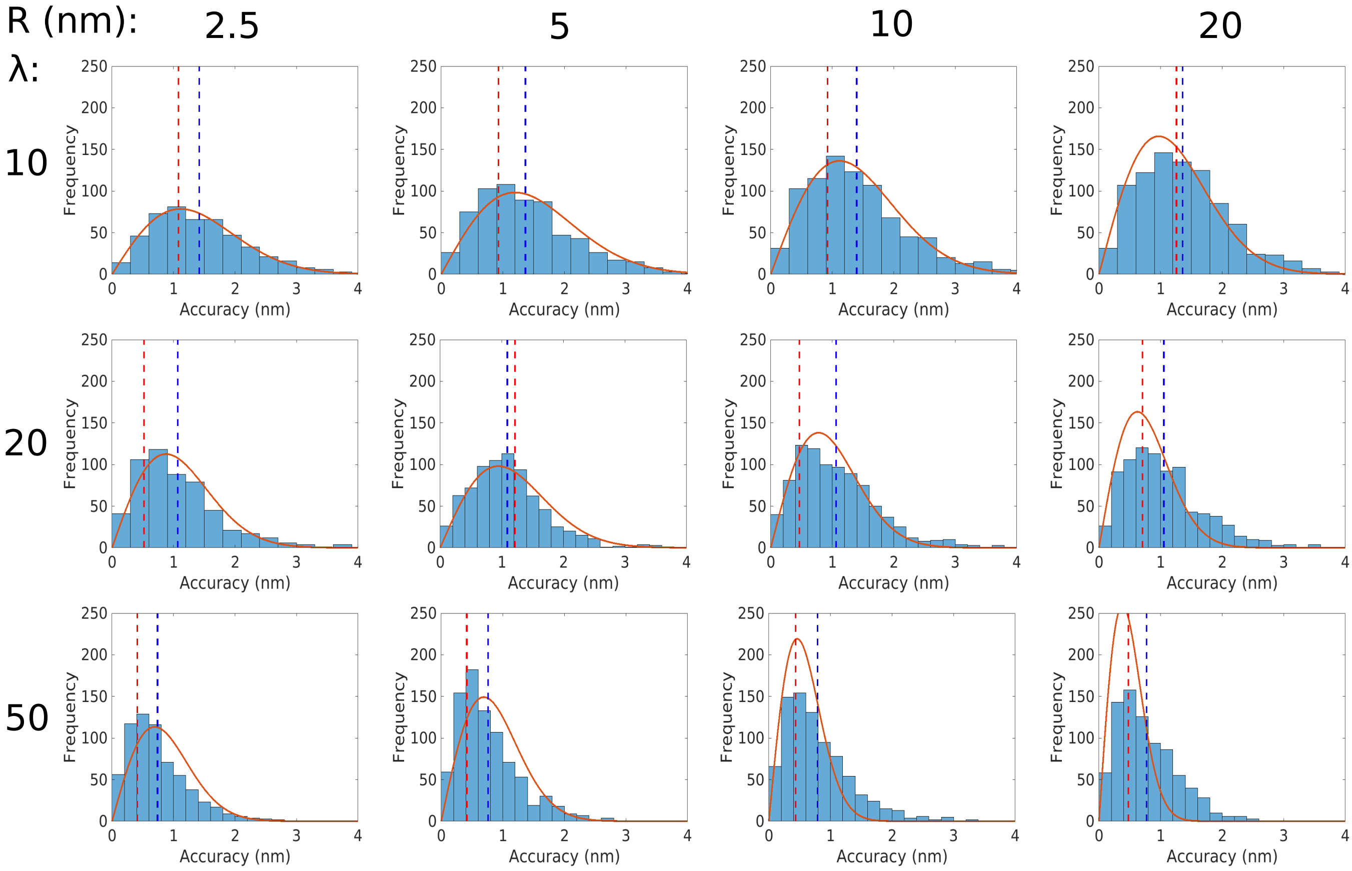

Supplementary Figure 7: Histogram of accuracies (for $N = 100$ 8-mer structures similar to the MAPN images in Supplementary Fig. 4), which is the distance between the real and found emitter positions. The red and blue dashed lines, respectively, show the mode and the mean of the histograms. The magenta curves are plots of $f(r) \sim r \exp\left(-r^2/2\sigma^2\right)$, where the parameter σ was set to the corresponding precision means in Supplementary Fig. 6. In addition, the areas under the curves for the displayed data ranges were adjusted to match the corresponding histogram areas.

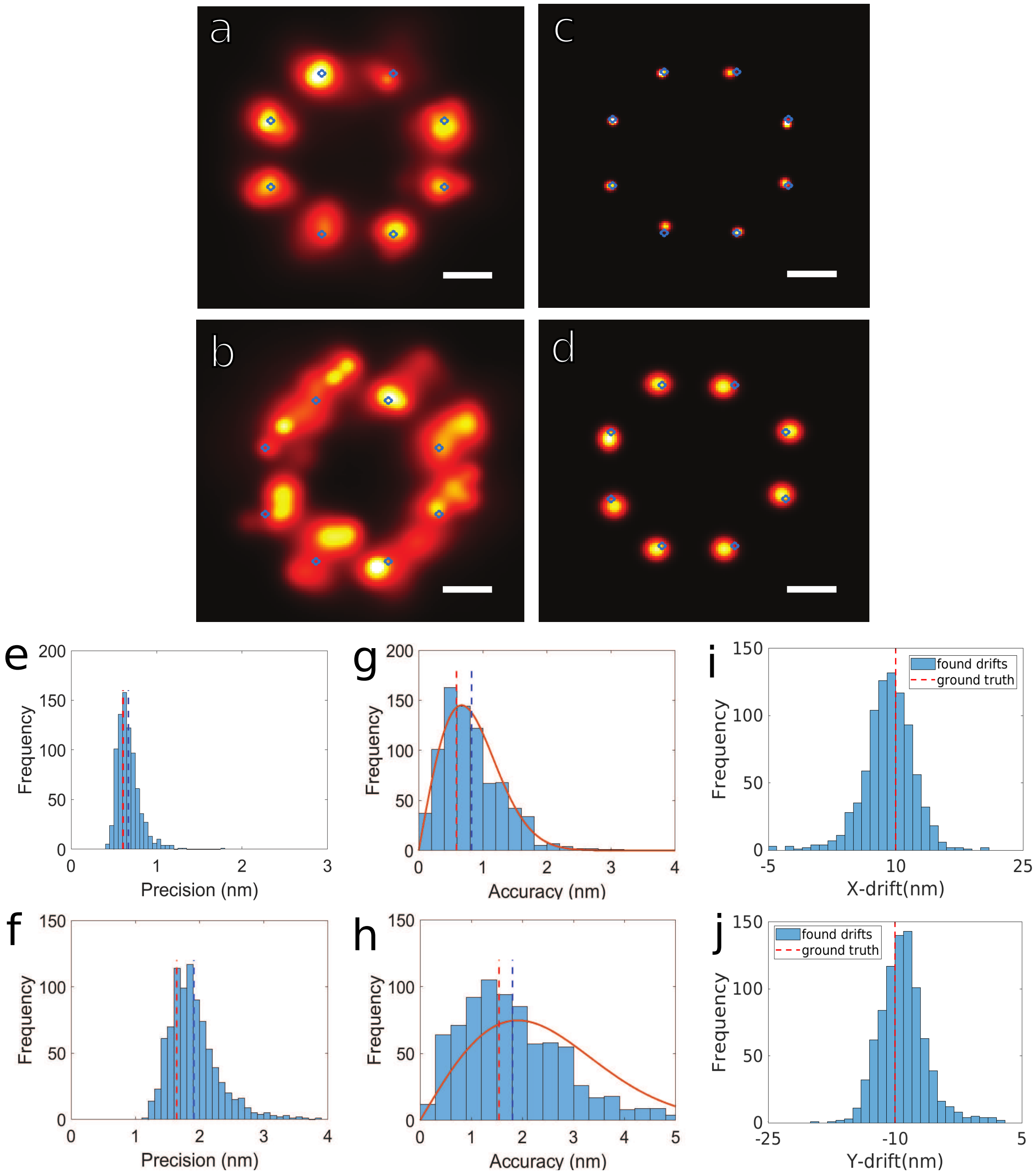

Supplementary Figure 8: Impact of emitter drifts on precision. (a-b) The emitters in panel a have no movement, while emitters in panel b have (10,-10) nm drift, respectively, in x and y directions over the entire 10,000 frames (Note that the origin of location for the images is the top left corner). The average intensity of binding/blinking events, PSF size, radius of the 8-mers and the average number of blinking/binding events per emitter were 1800 photons, 120 nm, 20 nm and 50, respectively. (c-d) BaGoL MAPN images for the static and drifted emitters in panels a and b, respectively. BaGoL analysis was repeated for $N = 100$ data sets used in panels a-d. (e-f) Histograms of resulting precisions for the static and drifted emitters in panels a and b, respectively. (g-h) Histograms of resulting accuracies for the static and drifted emitters in panels a and b, respectively. (i-j) Histograms of found total drifts along x and y directions by BaGoL for the drifted emitters in panel b. The blue circles show true emitter positions. The histograms were produced using $N = 100$ 8-mers for both drifted and static emitters. Scale bars are 10 nm.

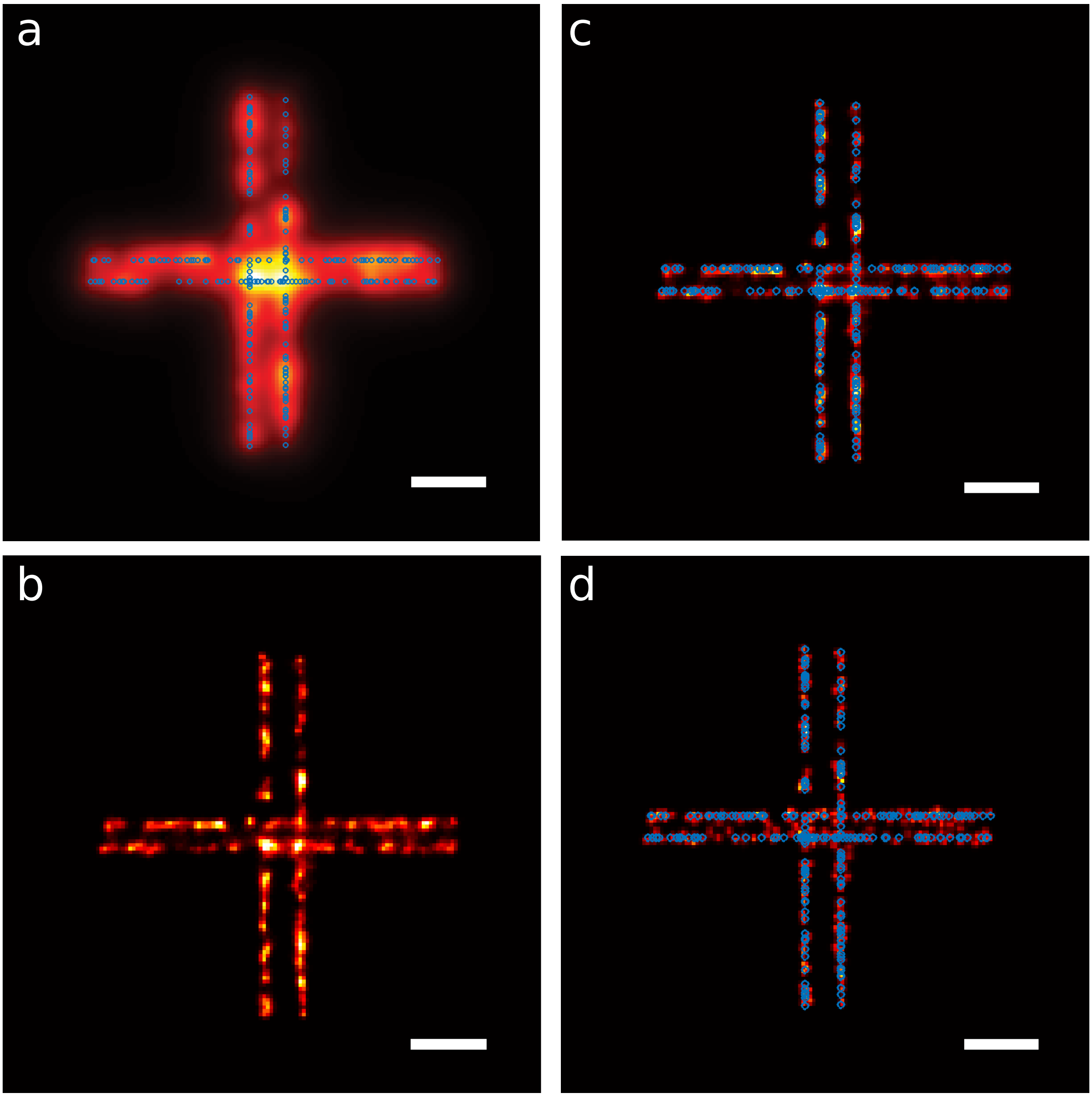

Supplementary Figure 9: Analysis of continuous lines of emitters. (a) Super-resolution image overlaid with true emitter positions, (b) posterior image obtained using a Poisson prior for number of emitters, (c) posterior image overlaid with true positions, (d) MAPN image overlaid with true emitter positions. Blue circles show true emitter positions. To simulate this data, 70 emitters were randomly placed on each line of length 100 nm. The horizontal and vertical lines are separated by 6 nm and 12 nm, respectively. The average intensity, PSF size and mean number of localizations per emitter were, respectively, 1800, 120 nm and 50. BaGoL analysis was repeated for $N = 2$ similar data sets. Scale bars are 20 nm.

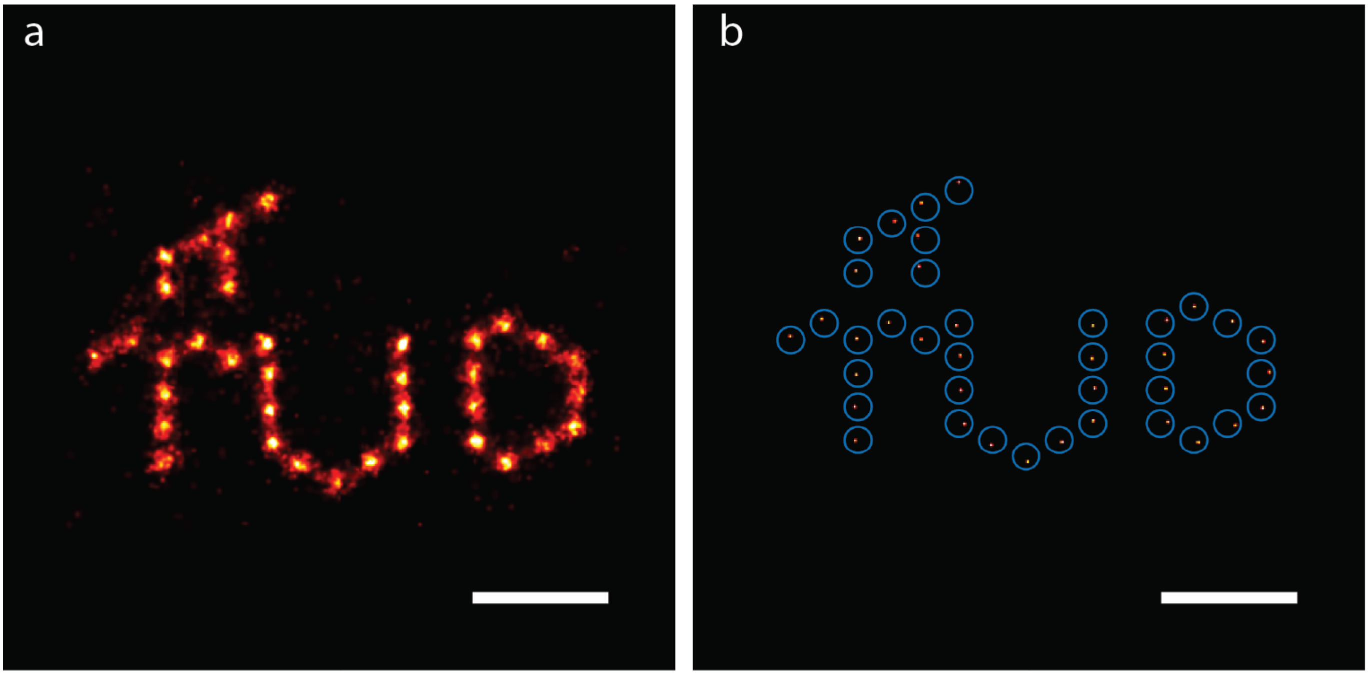

Supplementary Figure 10: Bayesian Grouping of Localizations applied to aligned structures. (a) The MAPN results of multiple structures were aligned with a template and combined. (b) The MAPN image of applying BaGoL to the collection of MAPN results shown in (a). The true emitter positions (template) are shown with blue circles with radii of 2 nm. This figure was made using $N = 170$ identical structures selected from a data set. The source data is included within this paper. The scale bars are 20 nm

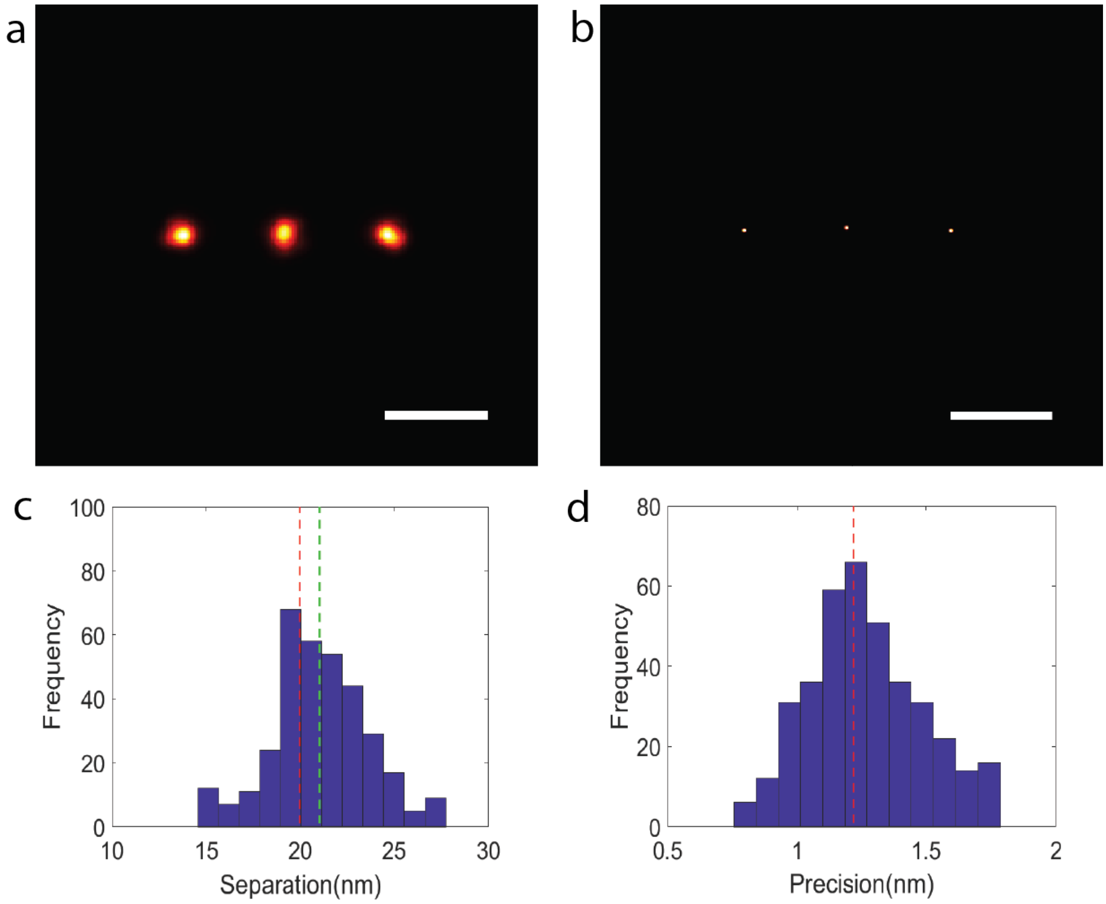

Supplementary Figure 11: The separation and precision for Gattaquant DNA-rulers. (a) Collection of aligned MAPN results from multiple DNA-rulers. (b) The MAPN result applying BaGoL to the collection of aligned MAPN coordinates in (a). Scale bars are 20 nm. (c) The histogram of separations between the emitters from 150 DNA-rulers. The red line shows 20 nm separation and the green line represents the found separation from (b), 20.7 nm. (d) The precisions for the MAPN localizations from 150 DNA-rulers. This figure was made using $N = 50$ identical structures selected from a data set. The source data is included within this paper.

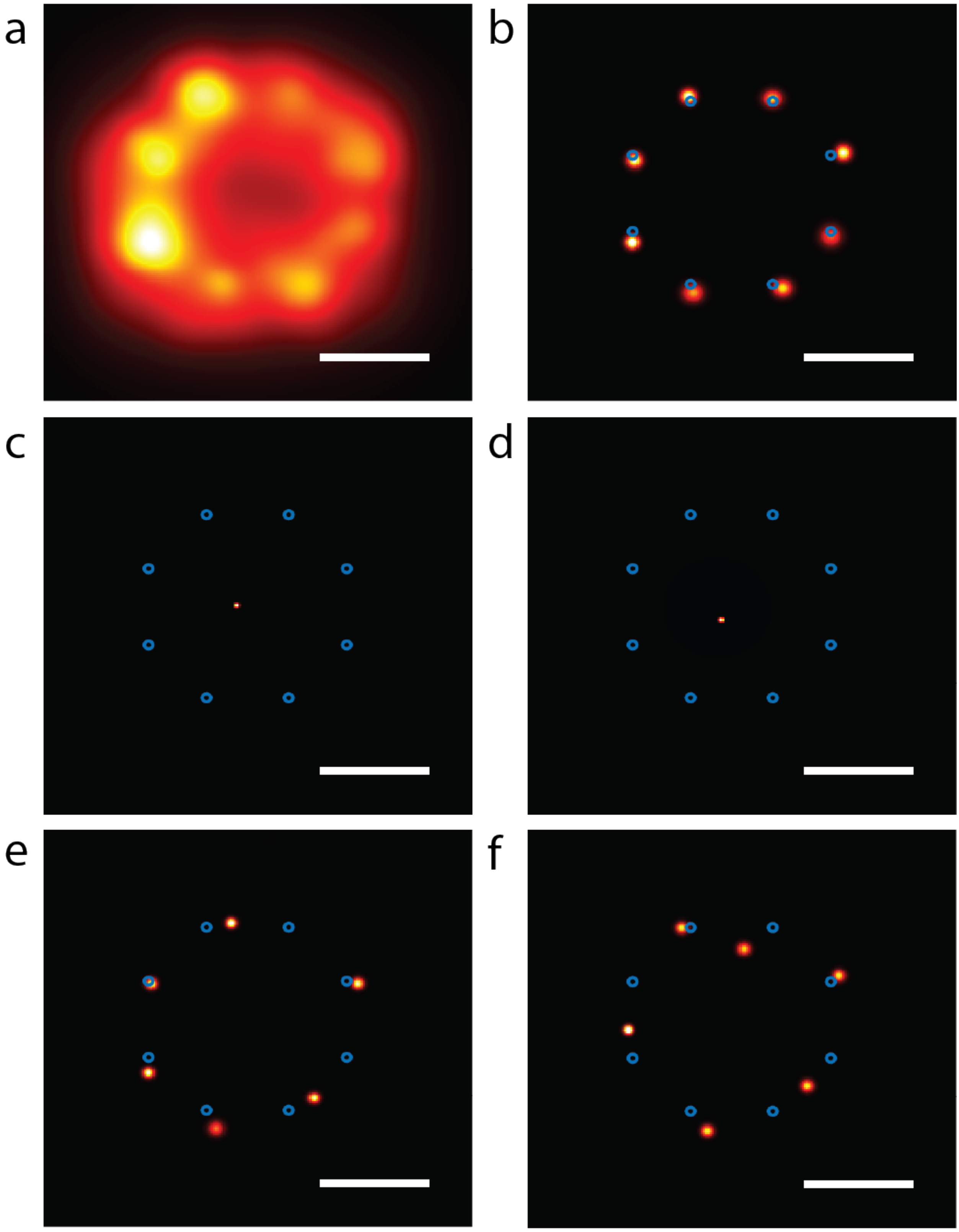

Supplementary Figure 12: Comparison of BaGoL to other clustering algorithms. Eight emitters were evenly spaced on a ring with radius of 10 nm, an expected $\lambda = 50$ blinking/binding events per emitter, and an average of 1800 photons per event. (a) Synthetic super-resolution image. (b) MAPN result from BaGoL. (c) Result from the DBSCAN algorithm with the maximum distance between the points within a cluster set to the mean localization precision and $\lambda - 2\sqrt{\lambda}$ as the minimum number of points within a cluster. (d) Outcome of the algorithm described in [2], where we used the recommended value, 20, for the Dirichlet prior and a gamma prior on the size of the clusters with average of 4 nm, which was the mean value of the localization precisions. (e) The best outcome of k-means from 10 different initializations with $K = 8$. (f) Gaussian Mixture Model result using 8 clusters. We obtain similar results to panels b-f using 3 other data sets similar to panel a. Scale bars are 10 nm.

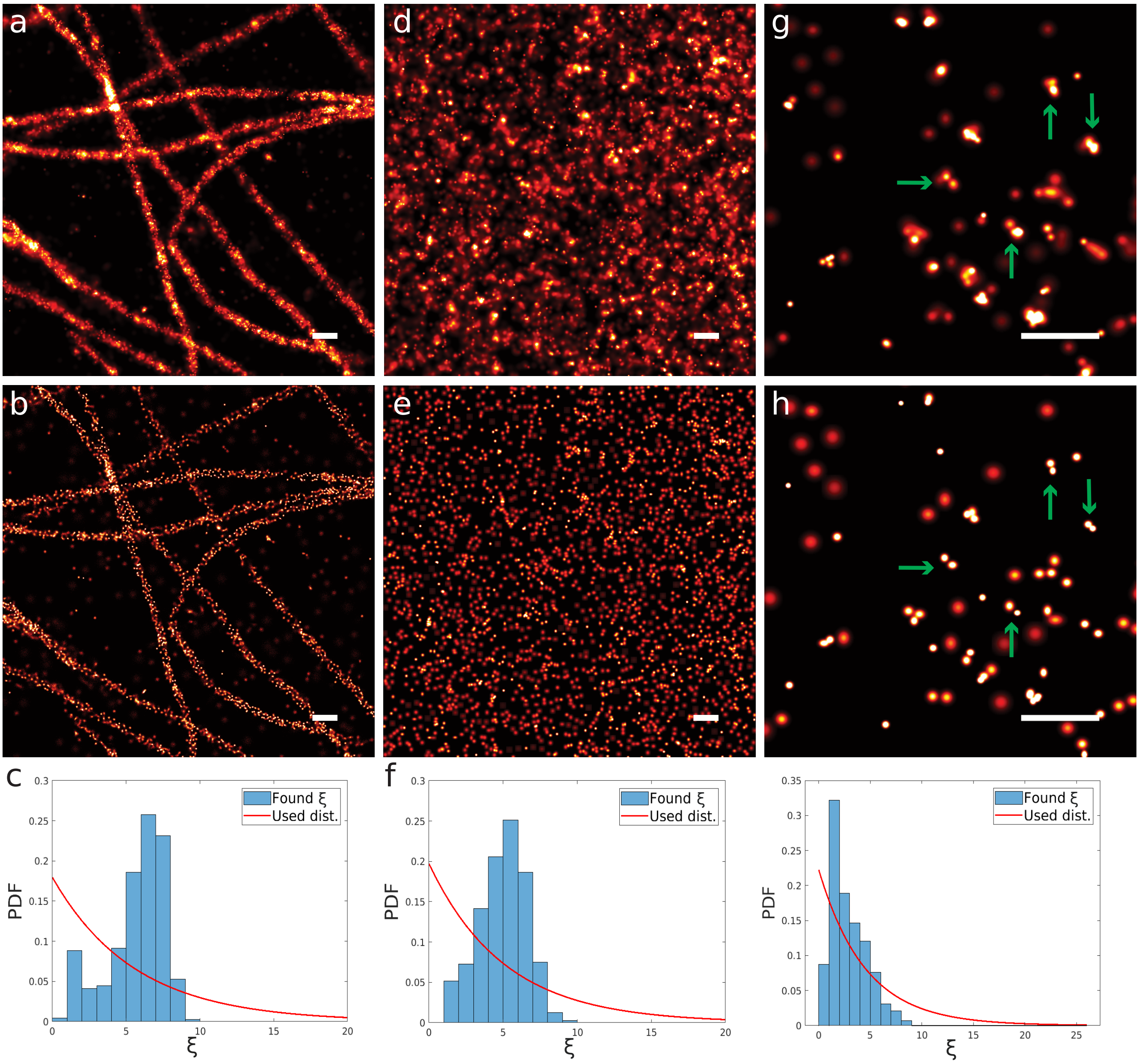

Supplementary Figure 13: BaGoL applied to experimental dSTORM data. (a) Traditional SR reconstruction of microtubules. (b) BaGoL posterior image of microtubules. (c) Used prior and found distribution of ξ, number of localizations per emitter, for microtubules. (d) Traditional SR reconstruction of CD82. (e) BaGoL MAPN image of CD82. (f) Used prior and found distribution of ξ for CD82. (g) Traditional SR reconstruction of EGFR. (h) BaGoL MAPN image of EGFR. (i) Used prior and found distribution of ξ for EGFR. The green arrows point to EGFR dimers. BaGoL analysis was repeated for 2, 2, and 4 regions similar to panels a, d, and g, respectively. PDF stands for probability density function. The source data are included within this paper. Scale bars are 200 nm.

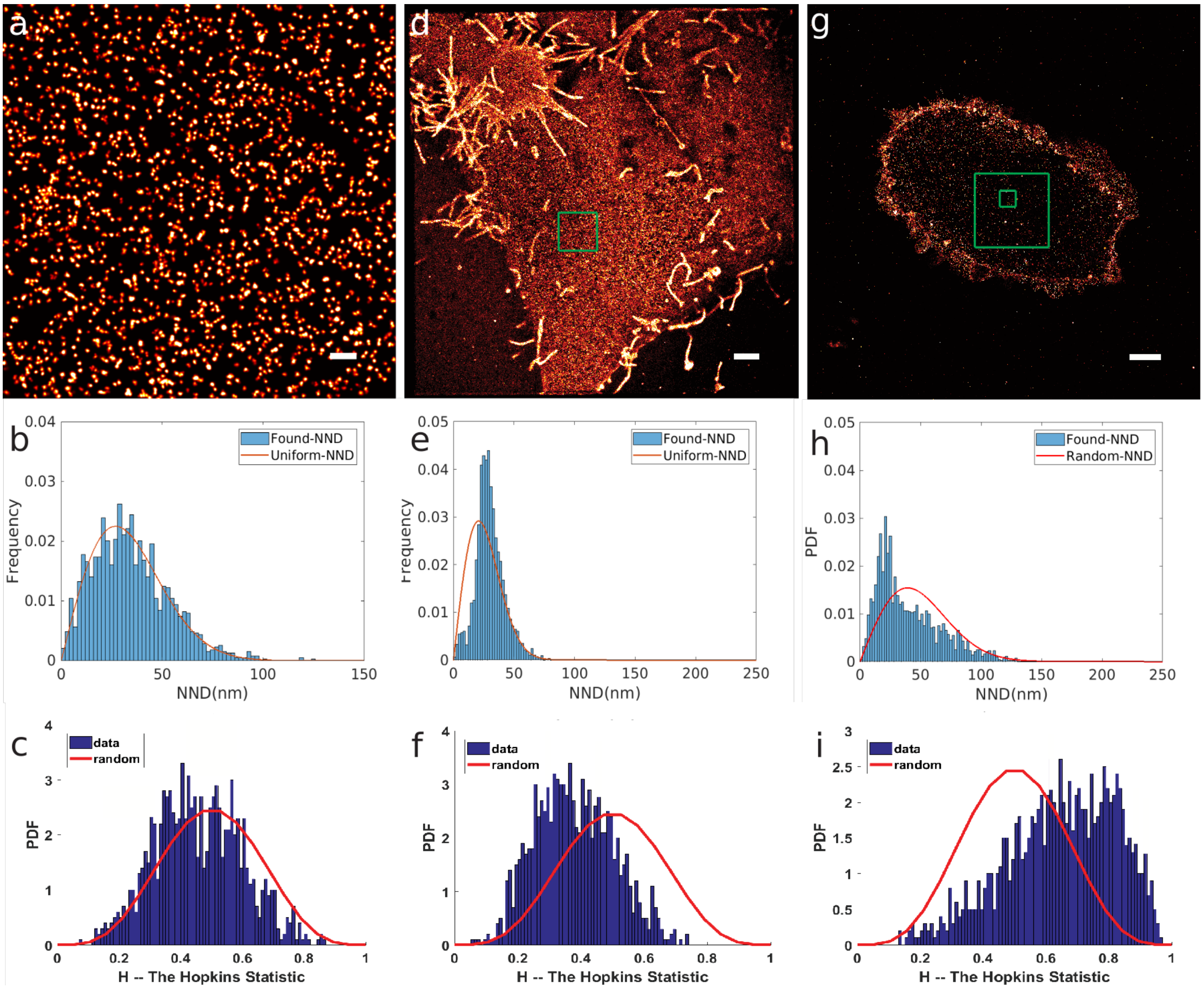

Supplementary Figure 14: Biological samples. (a) SR image of uniform randomly distributed simulated data with emitter density equal to that of CD82 (~220 μm^{-2}). The number of blinking events per emitter was taken from a Poisson distribution with an average of 8 where the mean intensity of blinking events was 400 photons and the PSF size was 120 nm. Scale bar is 200 nm. (b) Found nearest neighbor distribution (NND) of emitters in simulated data. (c) Hopkins' statistics for simulated data. (d) SR image of dSTORM data of CD82. The box indicates the region used for BaGoL analysis shown in Supplementary Fig. 13d. The scale bars are 2 μm. (e) Found NND of emitters in CD82 data. (f) Hopkins' statistics for CD82 data. (g) SR image of dSTORM data of EGFR. The larger box shows the region used for analysis. The smaller box is the zoomed in region shown in Supplementary Fig. 13g. Scale bar is 2 μm. (h) Found NND of emitters in EGFR data. (i) Hopkins' statistics for EGFR. We repeated the analyses for 2 different regions of the data sets in panels d and g. The source data is included within this paper.

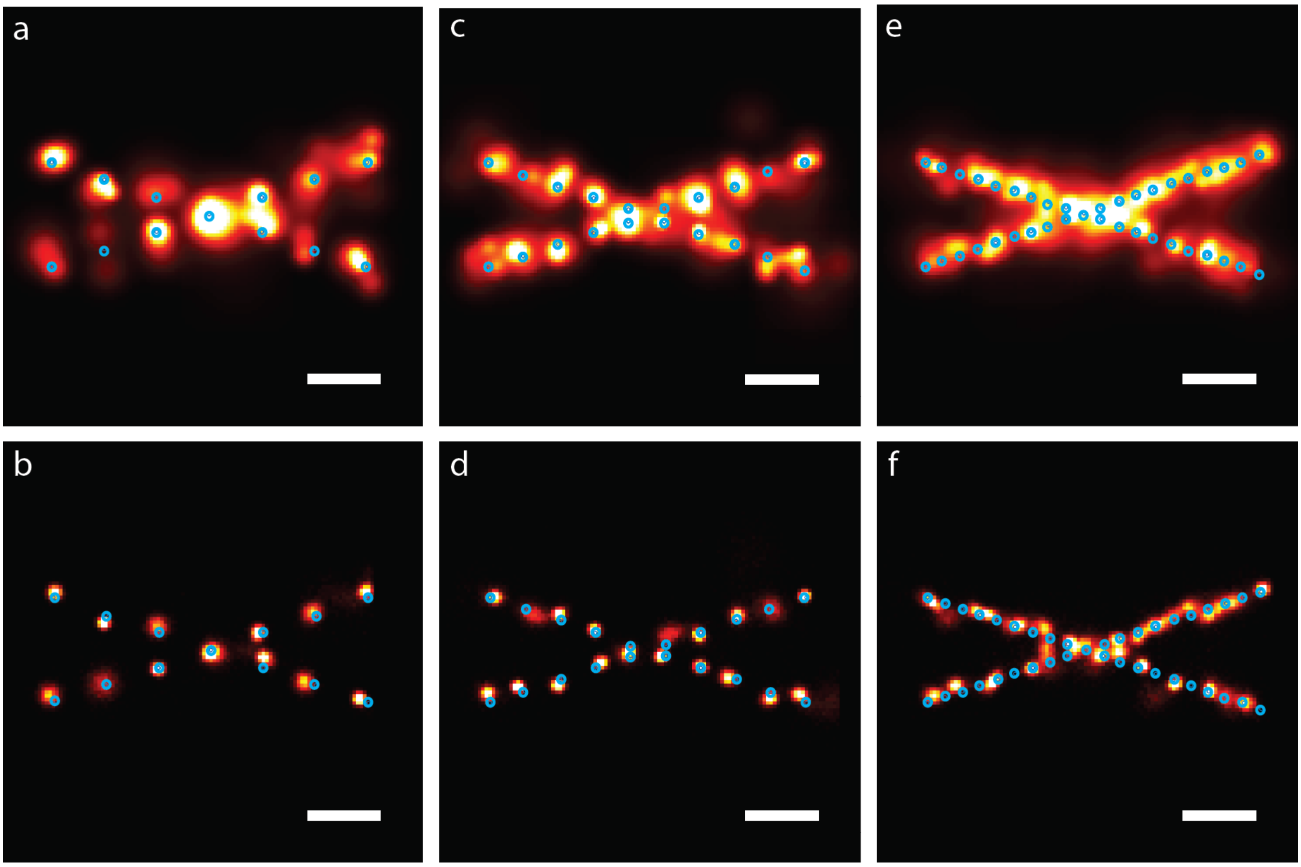

Supplementary Figure 15: BaGoL on synthetic dSTORM data in the presence of bleaching. To generate the raw SR data, the emitters were placed at equally spaced locations on the cross. There is considerable bleaching and the average number of blinking events before bleaching is 5. The average number of photons emitted in a blinking event is 1800. (a, c, e) SR images of cross with 15, 10 and 5 nm spacing between emitters, respectively. (b, d, f) Posterior images from BaGoL. Blue circles are centered on true emitter positions. BaGoL analysis was repeated for 4 structures similar to panels a, c, and e. The scale bars are 20 nm.

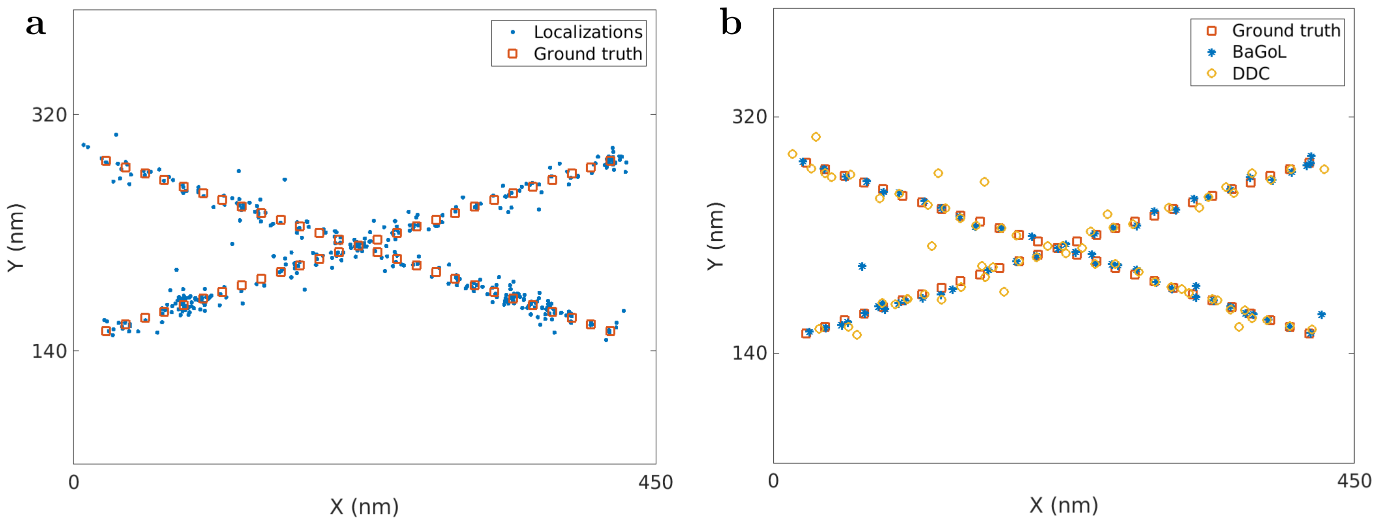

Supplementary Figure 16: BaGoL vs DDC [1]: synthetic dSTORM data. (a) Plot of synthesized localizations and true emitter locations. (b) Plot of found emitter locations by BaGoL (MAPN locations) and DDC. We used the results to calculate JAC and RMSE for both BaGoL and DDC as described in the methods section. The found JACs are, respectively, ≈0.85 and ≈0.80 for BaGoL and DDC. The found RMSEs are, respectively, ≈13.2 nm and ≈12.9 nm for DDC and BaGoL. To generate the data, we placed 54 emitters on equally spaced (15 nm) positions on a cross. We assumed a photokinetic model with 4 states as described in ref. [1]: 1) pre-activation state; 2) bright state; 3) dark state; 4) bleach state. Furthermore, we assumed an activation rate of 0.005/frame, $K_{\mathrm{on}} = 0.2$/frame, $K_{\mathrm{off}} = 0.5$/frame and $K_{\mathrm{b}} = 0.1$/frame with a frame exposure time of 10 ms. We assumed 1000 frames of data were acquired to assure bleaching of all the emitters. To perform the DDC analysis, we used $N = 160$ (frame difference at which steady state is reached), which was found as described in the DDC software package.

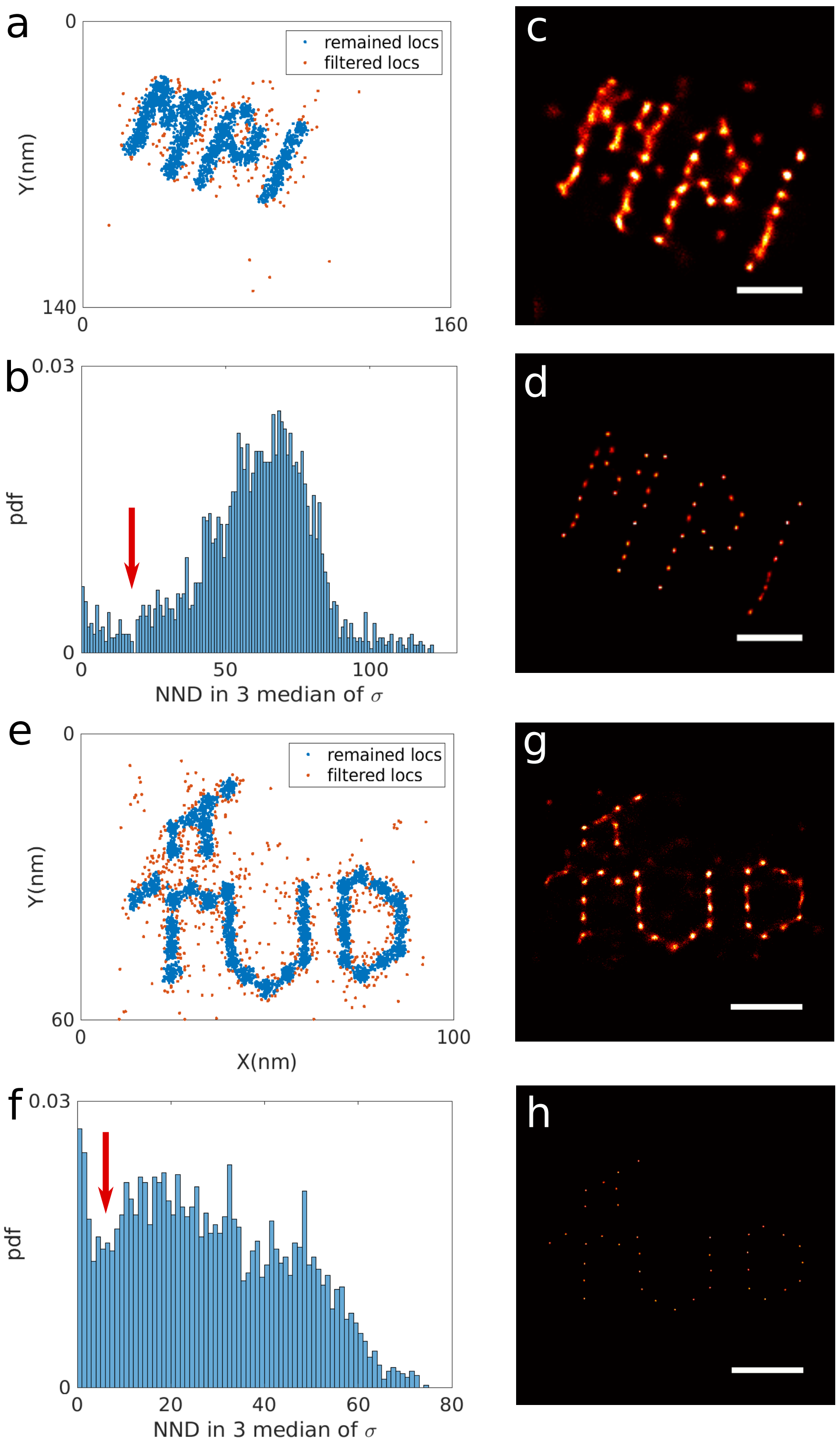

Supplementary Figure 17: NND filter for experimental DNA origami structures. (a) Plot of localizations for the MPI structure depicted in Fig. 2j-l. Red dots represent filtered localizations by the NND filter. (b) Histogram of the number of localizations within 3 time the median of localization precisions. (c) Posterior image of MPI from BaGoL without NND filter. (d) Posterior image of MPI using localizations after the NND filter (blue dots in panel a). (e) Plot of localizations for the TUD structure in Supplementary Fig. 10a. (f) Histogram of the number of localizations within 3 time the median of precisions. (g) Posterior image of the TUD structure from BaGoL without NND filter. (h) Posterior image of TUD using localizations after the NND filter (blue dots in panel e). The red arrows in panels b and f indicate the valley in the histograms. Localizations associated with the bars to the left of the red arrows are filtered out, shown by red dots in panels a and e. For the MPI and TUD structures, the used thresholding bar numbers were 24 and 12. The used thresholds are picked to be a little larger (by 5) than where the arrows indicate. The NND filter was applied to 3 and 1 structures similar to panels a and e, respectively. NND stands for nearest neighbor distribution. Scale bars are 20 nm.

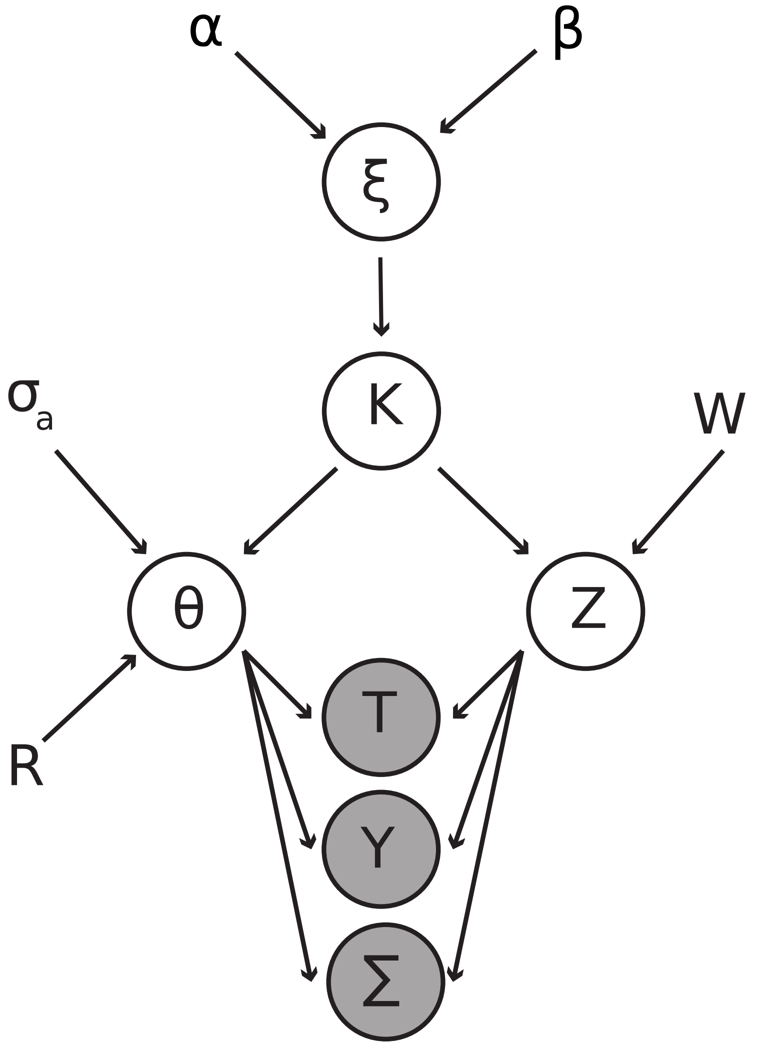

Supplementary Figure 18: Graph of the conditional dependence between random variables. Gray and blank circles, respectively, represent given data (set of localizations, Y, precisions, Σ, and times, T, of blinking events), and unknown parameters (allocation of localizations to emitters, Z, emitter positions (and drift velocities if modeled), θ, number of emitters, K, and prior on K (localization per emitter distribution), ξ). Prior hyper-parameters are shown without circles (the allowed range of emitter positions, R, the variance of drift velocities, σ_a^2, set of relative weights of emitters, W, and parameters of the hyper-prior on ξ, namely α and β).

Supplementary Note 1: Reversible Jump Markov Chain Monte Carlo

Reversible Jump Markov Chain Monte Carlo (RJMCMC) [4–7] is an extension of Markov Chain Monte Carlo (MCMC) [8,9] that allows sampling from parameter spaces with varying numbers of parameters, thus making inferences about the number of parameters as well as the parameters themselves. Here, we employ RJMCMC to develop a Bayesian grouping of localizations (BaGoL) algorithm. To do so, we use the following notation throughout the manuscript:

**Data:**	
N	Number of input localizations.
Y	Set of N input localizations, $Y = \{\vec{y}_1, ..., \vec{y}_N\}$.
Σ	Set of uncertainties/precisions corresponding to the given localizations, $\Sigma = \{\vec{\sigma}_1, ..., \vec{\sigma}_N\}$.
T	Set of times that the corresponding localizations were recorded, $T = \{t_1, ..., t_N\}$.
**Unknown parameters:**	
K	Number of underlying emitters that generated the localizations.
θ	Set of emitter positions and their corresponding drift velocities representing a generic model, $\theta = \{\vec{\mu_1}, \vec{a_1}, ...\}$.
Z	Allocation parameter which associates localizations to different emitters.
$\{.\}_{Z_i=j}$	Subset of input localizations allocated to the jth emitter.
N_j	Number of localizations allocated to the jth emitter.
**Prior and proposal distributions' parameters:**	
ξ	Hyper-parameter of prior on K, which can be either a scalar λ or a vector (γ, η).
λ	Average number of localizations per emitter used in Poisson prior on K.
γ	Scale parameter of gamma prior on K.
η	$K\eta$ is the shape parameter of gamma prior on K.
α	Shape parameter of gamma hyper-prior on ξ.
β	Scale parameter of gamma hyper-prior on ξ.
α_{prop}	Parameter of the gamma proposal distribution.
W	Set of relative weights of emitters, $W = \{w_1, ..., w_K\}$.
R^d	Range of localizations in the dth dimension.
σ_a^2	Variance of drift velocities.
**Indices:**	
i	Subscript referring to localizations.
j	Subscript referring to emitters.
d	Superscript referring to the spatial components of parameters.

BaGoL takes a set of localizations Y, the corresponding sets of precisions, Σ and time stamps T (times are only required when modeling drift) to make inference about the number of the emitters, K, and their positions, θ, by appropriate grouping and combining of the given localizations, Y. To accomplish this goal, BaGoL iteratively take samples from the posterior using 4 different types of jumps (Fig. 1). ***Birth*** explores the possibility of adding a new emitter to the current model. ***Death*** is the opposite of birth and examines the elimination of one of the existing emitters from the current model. ***Allocate*** directly samples the allocation of localizations to the emitters, Z [6]. ***Move*** updates the positions and movement parameters, θ, with fixed allocations using. In what follows, we first derive the likelihood model and the posterior. We next proceed to describe the sampling strategy and the jump types in more details.

1 Likelihood, Prior and Posterior

RJMCMC is often used in a Bayesian context to sample from the joint posterior of a system where both the number of parameters and the parameters themselves are random variables. Here, we describe the BaGoL's posterior.

1.1 Posterior

In our problem, the posterior, $\pi(K, Z, \theta|Y, \Sigma, T)$, is proportional to the product of the likelihood, $P(Y, \Sigma, T|Z, \theta)$, and priors, $P(K|\xi), P(Z|K, W)$ and $P(\theta|K, R, \sigma_a)$. Considering the graph in Supplementary Fig. 18, the posterior can be written as follows

$$\pi(K, Z, \theta|Y, \Sigma, T) = \frac{P(Y, \Sigma, T|Z, \theta)P(K|\xi)P(Z|K, W)P(\theta|K, R, \sigma_a)}{P(Y, \Sigma, T)} \tag{1}$$

where $1/P(Y, \Sigma, T)$ is the constant of proportionality which is called the evidence.

1.2 Likelihood

Here, we begin by deriving the likelihood for a single localization, then extend it to a single emitter and finally describe the likelihood of a set of localizations from multiple emitters. The likelihood of the ith localization belonging to the jth emitter with position μ_j^d for the dth spatial component is given by

$$f(y_i^d, \sigma_i^d | \mu_j^d, Z_i = j) = \frac{1}{\sqrt{2\pi(\sigma_i^d)^2}} \exp\left[-\frac{(y_i^d - \mu_j^d)^2}{2(\sigma_i^d)^2}\right]. \tag{2}$$

The likelihood of the jth emitter given the set of localizations associated to this emitter is thus

$$f(\{y_i^d\}_{Z_i=j}, \{\sigma_i^d\}_{Z_i=j} | \mu_j^d) = \prod_{i|Z_i=j} f(y_i^d, \sigma_i^d | \mu_j^d, Z_i = j). \tag{3}$$

We allow independent movement for each emitter over time to accommodate factors such as residual drift, bad fixation, etc. Including these movements as a linear drift, likelihood (2) takes the form

$$f(y_i^d, \sigma_i^d, t_i | \mu_j^d, a_j^d, Z_i = j) = \frac{1}{\sqrt{2\pi(\sigma_i^d)^2}} \exp\left[-\frac{\left(y_i^d - \left(\mu_j^d + a_j^d t_i\right)\right)^2}{2(\sigma_i^d)^2}\right] \tag{4}$$

in which a_j^d is the dth component of the movement per frame (drift velocity) of the jth emitter. Taking into account these movements, the likelihood for the jth emitter takes the following form

$$f(\{y_i^d\}_{Z_i=j}, \{\sigma_i^d\}_{Z_i=j}, \{t_i\}_{Z_i=j} | \mu_j^d, a_j^d) = \prod_{i|Z_i=j} f(y_i^d, \sigma_i^d, t_i | \mu_j^d, a_j^d, Z_i = j) \tag{5}$$

At the end, the multi-emitter likelihood of a given data set can be written as

$$P(Y, \Sigma, T | Z, \theta) = \prod_{d=1}^{D} \prod_{i=1}^{N} f(y_i^d, \sigma_i^d, t_i | \theta, Z) = \prod_{i=1}^{N} f(\vec{y}_i, \vec{\sigma}_i, t_i | \theta, Z) \tag{6}$$

where D is number of the spatial components and $f(y_i^d, \sigma_i^d, t | \theta, Z)$ is given in (2) and (4) for the static and non-static emitters as appropriate.

1.3 Prior Distributions

To obtain the posterior, we need to include priors over the unknown parameters. The prior on the number of emitters is calculated assuming either a Poisson or a gamma distribution of localizations from each emitter with a mean number of localizations per emitter, λ. Given a total number of observed localizations N, the prior is then

$$\begin{aligned} P(K|\xi) &= \text{Poisson}(N, K\lambda) / \sum_K \text{Poisson}(N, K\lambda), \text{ or} \\ P(K|\xi) &= \text{gamma}(N, K\eta, \gamma) / \sum_K \text{gamma}(N, K\eta, \gamma) \end{aligned} \tag{7}$$

where γ is the scale parameter and $\eta\gamma = \lambda$. The prior on allocations is taken to be a categorical distribution with the same weights for all the emitters, $\mathcal{C}(Z_i; [w_1, ..., w_K])$, where $w_1 = ... = w_K = 1/K$

$$P(Z|K, W) = \prod_{i=1}^{N} \mathcal{C}(Z_i; [w_1, ..., w_K]) = \left(\frac{1}{K}\right)^N \tag{8}$$

and w_j is the relative weight of the jth emitter. The prior on the emitter positions is taken as a uniform distribution over the range of the data in each dimension, R^d.

$$P(\mu_j^d | R^d) = \frac{1}{R^d} \tag{9}$$

The prior on the drift parameters is taken as a normal distribution

$$P(a_j^d | \sigma_a) = \mathcal{N}\left(a_j^d; 0, \sigma_a^2\right) \tag{10}$$

where σ_a^2 is the variance of the drift velocities.

2 Sampling Strategy and Jump Types

Our sampling strategy includes four types of jumps, *i.e.*, procedures to propose new parameter values, used by BaGoL to iteratively sample the posterior: two intra-model jumps called ***Allocate*** and ***Move***, and two complimentary inter-model jumps called ***Birth*** and ***Death***. The intra-model jumps allow the exploration of the parameters of the current model, which are the emitter positions, drift velocities, and allocation of the localizations to the current emitters. The inter-model jumps let the chain to move between models with different numbers of emitters.

2.1 Proposing a Jump

At each step in the chain, *i.e.*, iteration, we pick a random jump type using the given occurrence probabilities for jumps, which we show by $P_{\text{Allocate}}, P_{\text{Move}}, P_{\text{Birth}}$ and P_{Death}, respectively, for the probabilities of proposing an ***Allocate***, a ***Move***, a ***Birth*** and a ***Death*** (for instance, $P_{\text{Allocate}} = 0.25, P_{\text{Move}} = 0.25, P_{\text{Birth}} = 0.25, P_{\text{Death}} = 0.25$). Note that we have

$$P_{\text{Allocate}} + P_{\text{Move}} + P_{\text{Birth}} + P_{\text{Death}} = 1. \tag{11}$$

Each jump then is accepted or rejected as specified for each jump below.

2.2 Intra-model jumps

Move

In this case, due to the closed form of the full conditional posterior eq. (12), the values for emitter positions and their drift velocities (if used) are modified by directly sampling from the full conditional posterior of θ, which is proportional to the likelihood and the prior on drift velocities and has the form of a multivariate normal distribution

$$\begin{aligned} P(\theta|K, Z, Y, \Sigma, T) &\propto P(Y, \Sigma, T|Z, \theta) \prod_j \prod_d P(\mu_j^d|R^d) P(a_j^d|\sigma_a) \\ &\propto \prod_j \prod_d \mathcal{N}\left(\left(\hat{\mu}_j^d, \hat{a}_j^d\right), \Xi_j^d\right) \end{aligned} \tag{12}$$

where $\mathcal{N}(.)$ represents the multivariate normal distribution with the center/maximum $(\hat{\mu}_j^d, \hat{a}_j^d)$ given in eq. (33), and the covariance matrix Ξ_j^d given in eq. (31). Note that the prior on emitter positions is a uniform distribution. During a ***Move***, all (μ_j^d, a_j^d) pairs are updated simultaneously assuming fixed K, Z.

Allocate

Here, again due to the closed form of the full conditional posterior eq. 13, allocations of localizations to emitters are updated by sampling from the full conditional distribution given all the other parameters

$$P(Z|K, \theta, Y, \Sigma, T) \propto P(Y, \Sigma, T|Z, \theta) P(Z|K, W) \propto P(Y, \Sigma, T|Z, \theta) \tag{13}$$

and always accepting the proposed allocations Z'. Note that the relative weights of emitters are considered the same in prior (8) and therefore the posterior is proportional to the likelihood.

Hierarchical Bayes

The hierarchical Bayes framework [13] allows learning the prior parameters by including hyper-priors on these parameters. Here, since it might be difficult to pre-calibrate the distribution of number of localizations per emitter, we use a hierarchical Bayes scheme to learn this distribution. To do so, we assume the distribution of the number of localizations per emitter is either a Poisson or gamma distribution; see SI section 1.3. In this scheme, the prior on the number of emitters (7) is used as a likelihood and we place a gamma prior on the parameters to guarantee non-negative values. We also use a gamma proposal distribution to guarantee positive proposed values.

Here, since the posterior does not have a standard close form, we use Metropolis-Hasting (MH) to calculate an acceptance ratio for the proposed values. The acceptance ratio is given by the ratio of the posteriors for the proposed and current values. Moreover, the proposal distribution must be included in the acceptance ratio due to its asymmetric nature. The acceptance ratios for both Poisson and gamma cases are derived below.

For the Poisson case, new λ values are proposed as

$$\lambda' \sim \text{gamma}\left(\alpha_{\text{prop}}, \frac{\lambda}{\alpha_{\text{prop}}}\right), \tag{14}$$

where prime indicates proposed values. The proposed value is then accepted by the probability of

$$A_\lambda = \frac{\text{Poisson}(N, K\lambda')\text{gamma}\left(\lambda', \alpha, \beta\right)\text{gamma}\left(\lambda', \alpha_{\text{prop}}, \frac{\lambda}{\alpha_{\text{prop}}}\right)}{\text{Poisson}(N, K\lambda)\text{gamma}\left(\lambda, \alpha, \beta\right)\text{gamma}\left(\lambda, \alpha_{\text{prop}}, \frac{\lambda'}{\alpha_{\text{prop}}}\right)}. \tag{15}$$

For the gamma prior, new values for η and γ are drawn in a similar fashion

$$\eta' \sim \text{gamma}\left(\alpha_{\text{prop}}, \frac{\eta}{\alpha_{\text{prop}}}\right), \tag{16}$$

$$\gamma' \sim \text{gamma}\left(\alpha_{\text{prop}}, \frac{\eta}{\gamma_{\text{prop}}}\right). \tag{17}$$

The acceptance ratios of the proposed values are given by

$$A_\eta = \frac{\text{gamma}(N, K\eta', \gamma)\text{gamma}\left(\eta', \alpha, \beta\right)\text{gamma}\left(\eta', \alpha_{\text{prop}}, \frac{\eta}{\alpha_{\text{prop}}}\right)}{\text{gamma}(N, K\eta, \gamma)\text{gamma}\left(\eta, \alpha, \beta\right)\text{gamma}\left(\eta, \alpha_{\text{prop}}, \frac{\eta'}{\alpha_{\text{prop}}}\right)}, \tag{18}$$

$$A_\gamma = \frac{\text{gamma}(N, K\eta, \gamma')\text{gamma}\left(\gamma', \alpha, \beta\right)\text{gamma}\left(\gamma', \alpha_{\text{prop}}, \frac{\gamma}{\alpha_{\text{prop}}}\right)}{\text{gamma}(N, K\eta, \gamma)\text{gamma}\left(\gamma, \alpha, \beta\right)\text{gamma}\left(\gamma, \alpha_{\text{prop}}, \frac{\gamma'}{\alpha_{\text{prop}}}\right)}. \tag{19}$$

2.3 Inter-model jumps

Birth and ***Death*** are complementary reversible processes. They allow proposing new models by the addition and removal of an emitter to/from the current model. The proposed jumps are accepted with the probability

$$\alpha = \min\{1, A\} \tag{20}$$

where

$$A = \frac{\pi(K', Z', \theta'|Y, \Sigma, T)}{\pi(K, Z, \theta|Y, \Sigma, T)} \frac{r'_m}{r_m} \frac{q'(u')}{q(u)} \left| \frac{\partial(\theta', Z', u')}{\partial(\theta, Z, u)} \right| \tag{21}$$

and the prime indicates the proposed parameters. The first term is the ratio of the posteriors. r'_m and r_m are the probabilities of proposing a jump and its complementary jump. $q(u)$ and $q'(u')$ are the proposal distributions used to generate the parameters u and u', respectively. The parameter u is a set of auxiliary parameters used together with the parameters of the current model (θ, Z) to generate the parameters of the proposed model (θ', Z'), and u' is used in the inverse process. The last term is the Jacobian of the transformation from (θ, Z, u) to (θ', Z', u').

Birth

Birth proposes a new model with an extra emitter. The position of the new emitter is sampled from the normalized SR-image, P_{Im}, and drift velocity of a new emitter is selected by drawing a sample from the prior on these parameters. Since there is a new emitter, we need to reallocate the localizations to this new set of emitters using eq. (13). The proposed model is accepted with probability given by eq. (20), where the posterior ratio is given by the likelihood ratio and prior ratios

$$\frac{\pi(K+1, Z', \theta'|Y, \Sigma, T)}{\pi(K, Z, \theta|Y, \Sigma, T)} = \frac{\prod_{i=1}^{N} f(\vec{y}_i, \vec{\sigma}_i, t_i|\theta', Z')}{\prod_{i=1}^{N} f(\vec{y}_i, \vec{\sigma}_i, t_i|\theta, Z)} \frac{\prod_{j=1}^{K+1}\prod_{d=1}^{D} \frac{1}{R^d}\mathcal{N}\left(a_j'^d; 0, \sigma_a^2\right)}{\prod_{j=1}^{K}\prod_{d=1}^{D} \frac{1}{R^d}\mathcal{N}\left(a_j^d; 0, \sigma_a^2\right)} \frac{P(Z'|K+1, W)}{P(Z|K, W)} \frac{\text{P}(K+1|\xi)}{\text{P}(K|\xi)} \tag{22}$$

and the ratio of proposal distributions is

$$\frac{q'(u')}{q(u)} = \frac{P_{\text{alloc}}(Z)}{P_{\text{alloc}}(Z')} \frac{1}{\prod_{d=1}^{D} \frac{1}{R^d}\mathcal{N}\left(a_{K+1}'^d; 0, \sigma_a^2\right)} \tag{23}$$

where $P_{\text{alloc}}(Z')$ is the probability of proposing the allocation Z' [6], and $a_{K+1}'^d$ is the drift velocity of the new emitter along the dth spatial dimension. Note that in a reverse process, which is *death*, an emitter is eliminated and only a reallocation of localizations is required and therefore there is no term related to emitter position and drift in the top of eq. (23).

To derive P_{alloc}, we begin by considering the probability of allocating the ith localization to the jth emitter given by

$$P_{\text{alloc}}(Z_i = j) = \frac{w_{z_i} f(\vec{y}_i, \vec{\sigma}_i, t_i|\theta, Z_i = j)}{\sum_{j=1}^{K} w_j f(\vec{y}_i, \vec{\sigma}_i, t_i|\theta, Z_i = j)} \quad (24)$$

and therefore the probability of the complete set of allocations is

$$P_{\text{alloc}}(Z) = \prod_{i=1}^{N} \frac{w_{z_i} f(\vec{y}_i, \vec{\sigma}_i, t_i|\theta, Z_i = j)}{\sum_{j=1}^{K} w_j f(\vec{y}_i, \vec{\sigma}_i, t_i|\theta, Z_i = j)}. \quad (25)$$

Finally, the acceptance probability of this jump is simplified as

$$A = \frac{\mathrm{P}(K+1|\xi)}{\mathrm{P}(K|\xi)} \frac{\prod_{i=1}^{N} \sum_{j=1}^{K+1} f(\vec{y}_i, \vec{\sigma}_i, t_i|\theta', Z_i' = j)}{\prod_{i=1}^{N} \sum_{j=1}^{K} f(\vec{y}_i, \vec{\sigma}_i, t_i|\theta, Z_i = j)} \left(\frac{K}{K+1}\right)^N \frac{P_{\text{Birth}}}{P_{\text{Death}}} \frac{1}{\Lambda\, P_{\text{Im}}(\vec{\mu}_{\text{new}})}. \quad (26)$$

where Λ and $P_{\text{Im}}(\vec{\mu}_{\text{new}})$ are the area of the SR-image and the probability of selecting the given location, $\vec{\mu}_{\text{new}}$ for the new emitter, respectively. The Jacobian is calculated from the parameters whose dimensions are modified in the *Birth* jump, namely positions and drift velocities. Since the current emitter positions and drift velocities are held fixed and the position and drift for the new emitter are directly sampled from either the prior or proposal distributions, the Jacobian is equal to one.

Death

One of the existing emitters is selected at random and deleted from the model and the localizations are allocated across the remaining emitters as described in the *Birth* section above. The acceptance probability is computed as

$$\alpha = \min\{1, A^{-1}\} \quad (27)$$

where the ratio A is given by eq. (26).

Supplementary Note 2: Chain Setup and Generation

The chain is initialized to a state with the number of emitters $\sim N/\lambda$, and random emitter positions are selected from the position prior. The jumps described in Supplementary Note 1 are iteratively proposed within a loop. If jumps are accepted, Z', θ' (the proposed values) are recorded in the chain, otherwise Z, θ (the current values) are recorded. The chain is comprised of two parts. The beginning portion of the chain is called the burn-in when the chain has not yet settled to a stationary distribution. In the second portion, the chain is exploring the stationary posterior distribution; this portion of the chain is returned for further analysis.

Supplementary Note 3: Data Flow

Before processing the data using BaGoL, some pre-processing is required. The localizations are assumed to be frame connected across consecutive frames so that one input localization represents one blinking event. Next, the blinking/binding events that are too bright are considered outliers and removed from the inputs to BaGoL. The BaGoL algorithm has several stages. Data often is generated over a large field of view (e.g., a whole cell), whereas this method is designed to be applied to small clusters of emitters. First, the region of measurement containing all the localizations is split into smaller sub-regions. Neighboring sub-regions are overlapped to avoid edge artifacts. Second, the localizations that are considered spurious are filtered out using a nearest neighbor algorithm. A localization is considered signal when it has at least N neighbors within R distance. Parameters R and N are specified by the user and can be estimated by inspecting nearest neighbor distributions in the data. The localizations in each sub-region are then further separated into discrete clusters using the hierarchical clustering algorithm [11]. This algorithm is very fast, and has only one parameter (the maximum distance between localizations within a cluster). Each cluster is then processed by the core RJMCMC algorithm. The post-burn-in portion of the chain is returned for further analysis. The chains inside the overlapping areas are eliminated and the chains from the pre-clusters are stitched back together to obtain the posterior for the entire data. In addition, we extract the states with the most repeated model (MAPN) from the returned chain and use it to calculate the MAPN parameters [7]. The extracted states have the same number of emitters, but the emitters are ordered differently and their positions are also slightly altered in each state. Therefore, we employed k-means clustering to find the emitter coordinates from the collection of the positions within the extracted states. The mean number of localizations for an emitter was

calculated by averaging over the number of localizations allocated to that emitter in the extracted states. At the end, the list of MAPN parameters including emitter coordinates, their associated precisions, drift velocities and the average number of localizations allocated to every emitter are returned.

Supplementary Note 4: Improved Precision from the Combination of Localizations

In this section, we provide a theoretical demonstration for improvement of precision by combining the localizations. To do so, we start by considering the localization precision for the ith (a single) blinking/binding event. In practice, this precision is a function of the background and number of photons collected, and would be returned by the fitting algorithm. However, for the sake of illustration, we can approximate this as [12]

$$\sigma_i \approx \frac{\sigma_{\text{PSF}}}{\sqrt{I_i}} \tag{28}$$

where σ_{PSF} and I_i are the size of the point spread function (PSF) and the number of photons from the ith blinking/binding event. Now, assuming a set of localizations from an emitter, the likelihood is given by eq. (3). This likelihood can be used to calculate the precision $\sigma_{\mu_j^d}$ for the emitter position μ_j^d obtained via the combination of the localizations associated with this emitter:

$$\frac{1}{(\sigma_{\mu_j^d})^2} = \sum_{i|Z_i=j} \frac{1}{\left(\sigma_i^d\right)^2} \tag{29}$$

Substituting eq. (28) into eq. (29) yields

$$\sigma_{\mu_j^d} = \frac{\sigma_{\text{PSF}}}{\sqrt{\sum_{i|Z_i=j} I_i}} \sim \frac{\sigma_{\text{PSF}}}{\sqrt{< I > \lambda}} = \frac{\sigma}{\sqrt{\lambda}} \tag{30}$$

where $\lambda, < I >$ and σ, respectively, indicate the average number of blinking/binding events per emitter, average number of photons per blinking/binding event and the average localization precision obtained from the photons collected during a blinking/binding event $\left(\sigma = \sigma_{\text{PSF}}/\sqrt{< I >}\right)$. The denominator in Eq. (30) is the square root of the sum of all the photons emitted in every blinking/binding event associated to the jth emitter during data acquisition. Therefore, the combination of the blinking/binding events results in a better localization precision, by a factor of $1/\sqrt{\lambda}$.

Supplementary Note 5: Emitter Parameters and their Uncertainties

The posterior, q, of position and drift velocities of the jth emitter is proportional to the product of the likelihood (5) and the priors on position and drift velocity, respectively, given in (9) and (10). The posterior q takes the form of a multivariate normal distribution and can be employed to directly sample the emitter positions μ_j and drift velocities a_j as well as to calculate the theoretical precisions in the presence of the movements. The covariance matrix, Ξ_j^d, of this multivariate normal distribution is given by the inverse of $\mathbf{I}_j^d$

$$\Xi_j^d = (\mathbf{I}_j^d)^{-1} \tag{31}$$

where

$$\mathbf{I}_j^d = \begin{bmatrix} \frac{\partial^2 \log(q)}{\partial\left(\mu_j^d\right)^2} & \frac{\partial^2 \log(q)}{\partial \mu_j^d \partial a_j^d} \\ \frac{\partial^2 \log(q)}{\partial \mu_j^d \partial a_j^d} & \frac{\partial^2 \log(q)}{\partial\left(a_j^d\right)^2} \end{bmatrix} \tag{32}$$

The maximum of the multivariate normal distribution is given by

$$\hat{\mu}_j^d = \frac{A^d - a^d B^d}{C^d}, \quad \hat{a}_j^d = \frac{\sum_i \left(C^d y_i^d - A^d\right) / \left((\sigma_i^d)^2/t_i\right)}{C/\sigma_a^2 + \sum_i \left(C^d t_i - B^d\right) / \left((\sigma_i^d)^2/t_i\right)} \tag{33}$$

The square root of the diagonal elements of Ξ_j^d give the theoretical precisions

$$\sigma_{\mu_j^d}^2 = \frac{1/\sigma_a^2 + D^d}{C^d/\sigma_a^2 + C^d D^d - (B^d)^2}, \quad \sigma_{a_j^d}^2 = \frac{C^d}{C^d/\sigma_a^2 + C^d D^d - (B^d)^2} \tag{34}$$

where σ_a^2 is the variance of the prior on drift velocities and

$$A^d = \sum_i \frac{y_i^d}{\left(\sigma_i^d\right)^2},\ B^d = \sum_i \frac{t_i}{(\sigma_i^d)^2},\ C^d = \sum_i \frac{1}{(\sigma_i^d)^2},\ D^d = \sum_i \frac{t_i^2}{(\sigma_i^d)^2} \tag{35}$$

and the sum is over the localizations associated to the jth emitter. Note that the presence of emitter movements has a negative impact on the localization precision. This can be shown by comparing the moved emitter precision (34) with the static emitter precision (29)

$$\sigma_{\text{moved}} = \sqrt{\frac{1}{C - B^2/(1/\sigma_a^2 + D)}} \geq \sqrt{\frac{1}{C}} = \sigma_{\text{fixed}} \tag{36}$$

where the superscripts are dropped. Note that B, C and D are positive parameters.

Supplementary References

[1] Bohrer, Christopher H and Yang, Xinxing and Thakur, Shreyasi and Weng, Xiaoli and Tenner, Brian and McQuillen, Ryan and Ross, Brian and Wooten, Matthew and Chen, Xin and Zhang, Jin and others, "A pairwise distance distribution correction (DDC) algorithm to eliminate blinking-caused artifacts in SMLM",*Nature methods* **18**(6):669-677(2021).

[2] Rubin-Delanchy, Patrick, Garth L. Burn, Juliette Griffié, David J. Williamson, Nicholas A. Heard, Andrew P. Cope, and Dylan M. Owen, "Bayesian Cluster Identification in Single-Molecule Localization Microscopy Data", *Nature Methods* **12** (11): 1072–76 (2015).

[3] Smith, Carlas S., Nikolai Joseph, Bernd Rieger, and Keith A. Lidke, "Fast, Single-Molecule Localization That Achieves Theoretically Minimum Uncertainty", *Nature Methods* **7** (5): 373–75 (2010).

[4] Green, Peter J., "Reversible Jump Markov Chain Monte Carlo Computation and Bayesian Model Determination", *Biometrika* **82** (4): 711–32 (1995).

[5] Richardson, S. and Green, P. J., "On Bayesian analysis of mixtures with an unknown number of components", *J. R. Stat. Soc. Ser. B* **59**, 731-792 (1997).

[6] Viallefont, V., Richardson, S. and Green, P. J., "Bayesian Analysis of Poisson Mixtures", *Journal of Nonparametric Statistics* **14**: 1-2, 181-202 (2002).

[7] Fazel, Mohamadreza, Michael J. Wester, Hanieh Mazloom-Farsibaf, Marjolein B. M. Meddens, Alexandra S. Eklund, Thomas Schlichthaerle, Florian Schueder, Ralf Jungmann, and Keith A. Lidke., "Bayesian Multiple Emitter Fitting Using Reversible Jump Markov Chain Monte Carlo", *Scientific Reports* **9** (1): 13791 (2019).

[8] Metropolis, Nicholas, Arianna W. Rosenbluth, Marshall N. Rosenbluth, Augusta H. Teller, and Edward Teller. 1953. "Equation of State Calculations by Fast Computing Machines." The Journal of Chemical Physics 21 (6): 1087–92.

[9] Hastings, W. K., "Monte Carlo Sampling Methods Using Markov Chains and Their Applications." *Biometrika* **57** (1): 97–109 (1970).

[10] Geman, S. and Geman, D., Stochastic relaxation, Gibbs distribution and Bayesian restoration of images, *IEEE Transactions on Pattern Analysis and Machine Intelligence* **6**: 721-741 (1984).

[11] Everitt, Brian S., Sabine Landau, Morven Leese, and Daniel Stahl, "Cluster Analysis", Wiley Series in Probability and Statistics (2011).

[12] Ober, Raimund J., Sripad Ram, and E. Sally Ward, "Localization Accuracy in Single-Molecule Microscopy", Biophysical Journal 86 (2): 1185–1200 (2004).

[13] Bishop, Christopher M., "Machine Learning and Pattern Recognition", Springer (2006).
