# Supplementary material for: High-precision estimation of emitter positions using Bayesian grouping of localizations
Source: Nat Commun. 2022 Nov 22;13:7152. doi: 10.1038/s41467-022-34894-2 (PMC9684143; doi:10.1038/s41467-022-34894-2)
Supplement: Supplementary file 2 — Description of Additional Supplementary Files [file 41467_2022_34894_MOESM2_ESM.pdf]

File Name: Supplementary Movie 1:
Description:
Each thin circle represents a localization. Circle radii are equal to double of the corresponding localization precisions. Each thick red circle represents the current, inferred position of an emitter. Localizations of the same color are currently allocated to the same emitter. The animation shows the exploration of the possible number and position of emitters, as well as allocation of localizations to emitters.

File Name: Supplementary Movie 2:
Description:
Animation of the accumulation of the aligned MAPN coordinates of 170 identical TUD structures. Left panel displays addition of the new coordinate to the existing collection and the TUD structure becomes gradually more clear. Right panel represents the MAPN coordinates of a TUD structure to be added to the collection of aligned coordinates to the left.
Scale bar is 20 nm.

File Name: Supplementary Software 1
Description:
The software package contains code and example scripts for the Bayesian Grouping of Localizations (BaGoL) analysis method. The Zip-file contains an @folder, example scripts, data, a README text file, and a pre-compiled Windows mex executable needed for frame connection. The @folder contains a MATLAB class definition for the BaGoL algorithm. The README-file contains descriptions on how to run the example scripts.
