# Supplementary figures and images for: High-precision estimation of emitter positions using Bayesian grouping of localizations
Source: Nat Commun. 2022 Nov 22;13:7152. doi: 10.1038/s41467-022-34894-2 (PMC9684143; doi:10.1038/s41467-022-34894-2)

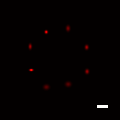

Supplement: Supplementary file 5 — Supplementary Software 1 [file 41467_2022_34894_MOESM5_ESM.zip › SoftwarePackage/Expected Results/Results_8mer/MAPN-SRImage.png]

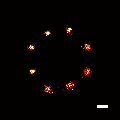

Supplement: Supplementary file 5 — Supplementary Software 1 [file 41467_2022_34894_MOESM5_ESM.zip › SoftwarePackage/Expected Results/Results_8mer/Posterior-SRImage.png]

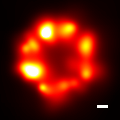

Supplement: Supplementary file 5 — Supplementary Software 1 [file 41467_2022_34894_MOESM5_ESM.zip › SoftwarePackage/Expected Results/Results_8mer/PreBaGoL-SRImage.png]

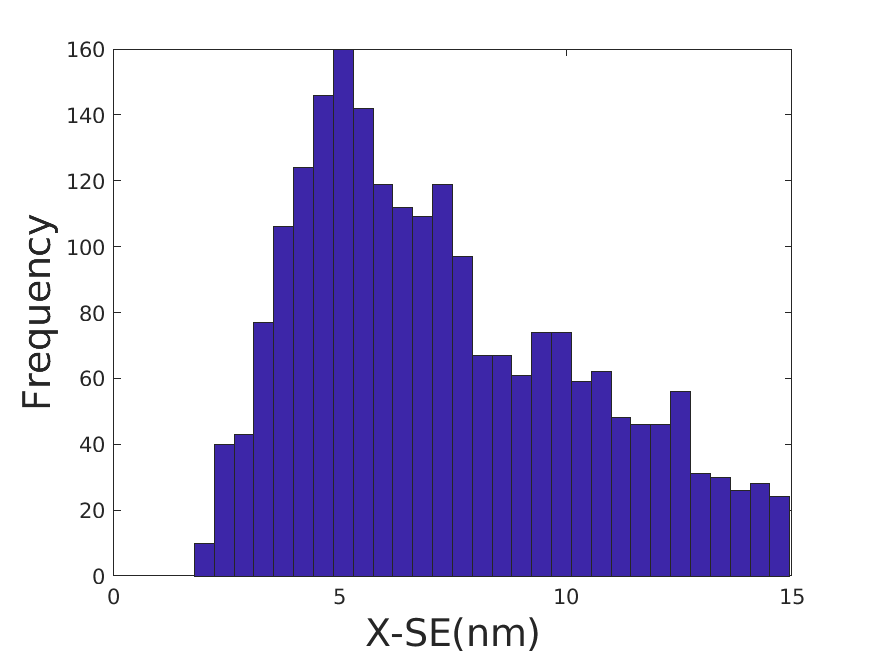

Supplement: Supplementary file 5 — Supplementary Software 1 [file 41467_2022_34894_MOESM5_ESM.zip › SoftwarePackage/Expected Results/Results_EGFR/BaGoL_X-SE.png]

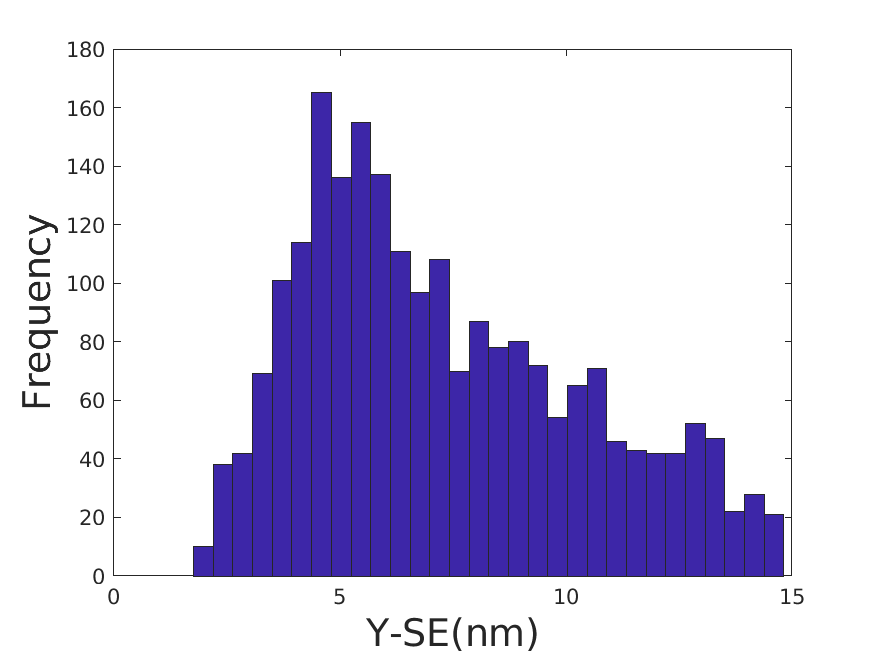

Supplement: Supplementary file 5 — Supplementary Software 1 [file 41467_2022_34894_MOESM5_ESM.zip › SoftwarePackage/Expected Results/Results_EGFR/BaGoL_Y-SE.png]

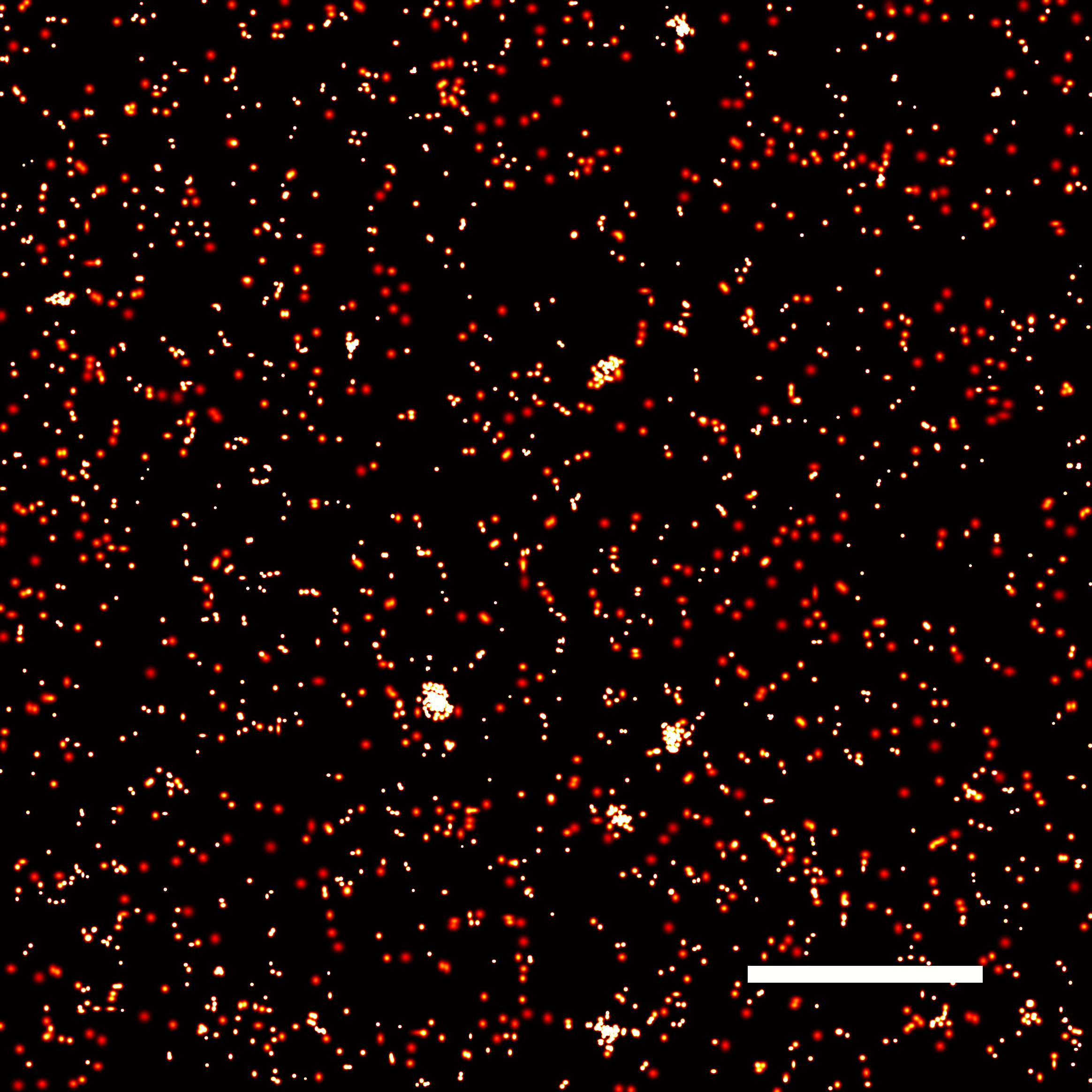

Supplement: Supplementary file 5 — Supplementary Software 1 [file 41467_2022_34894_MOESM5_ESM.zip › SoftwarePackage/Expected Results/Results_EGFR/MAPN-Im.png]

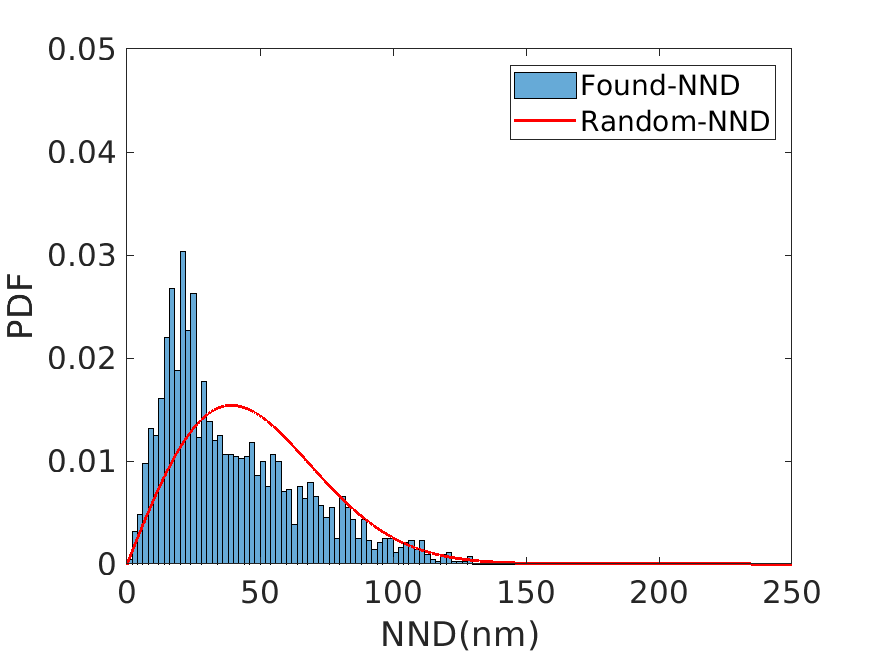

Supplement: Supplementary file 5 — Supplementary Software 1 [file 41467_2022_34894_MOESM5_ESM.zip › SoftwarePackage/Expected Results/Results_EGFR/NND-Hist+Random.png]

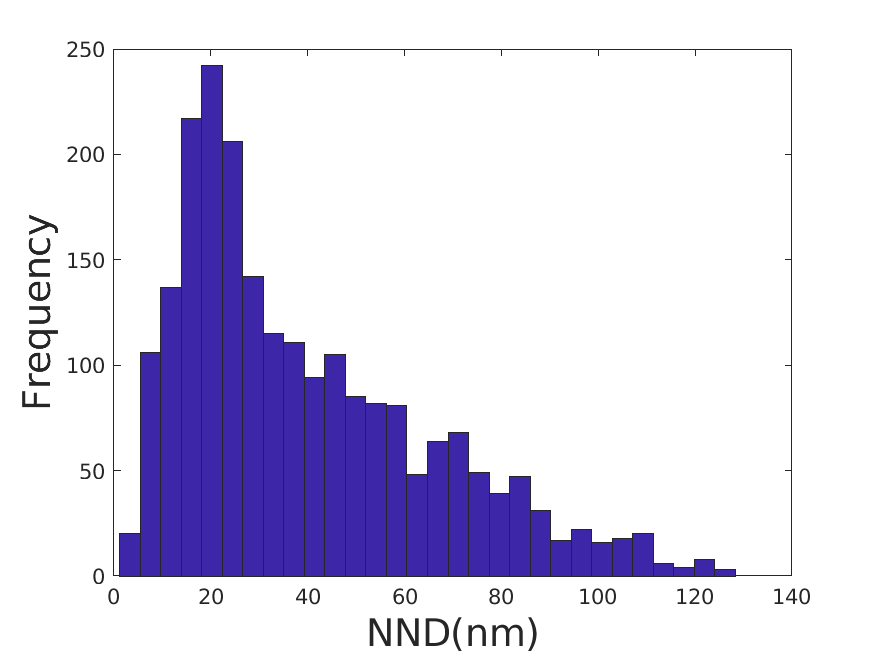

Supplement: Supplementary file 5 — Supplementary Software 1 [file 41467_2022_34894_MOESM5_ESM.zip › SoftwarePackage/Expected Results/Results_EGFR/NND.png]

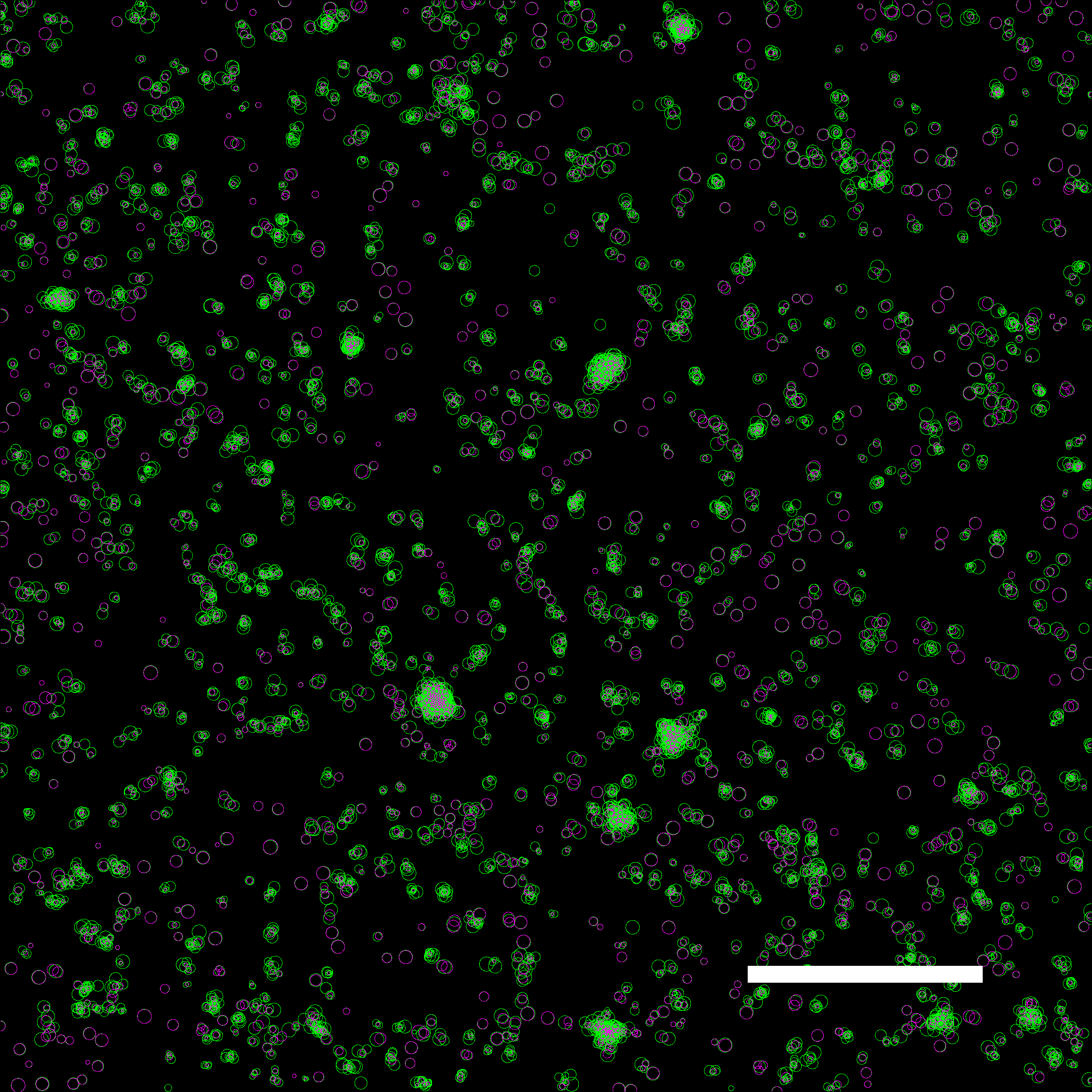

Supplement: Supplementary file 5 — Supplementary Software 1 [file 41467_2022_34894_MOESM5_ESM.zip › SoftwarePackage/Expected Results/Results_EGFR/Overlay_SR_Map_circle.png]

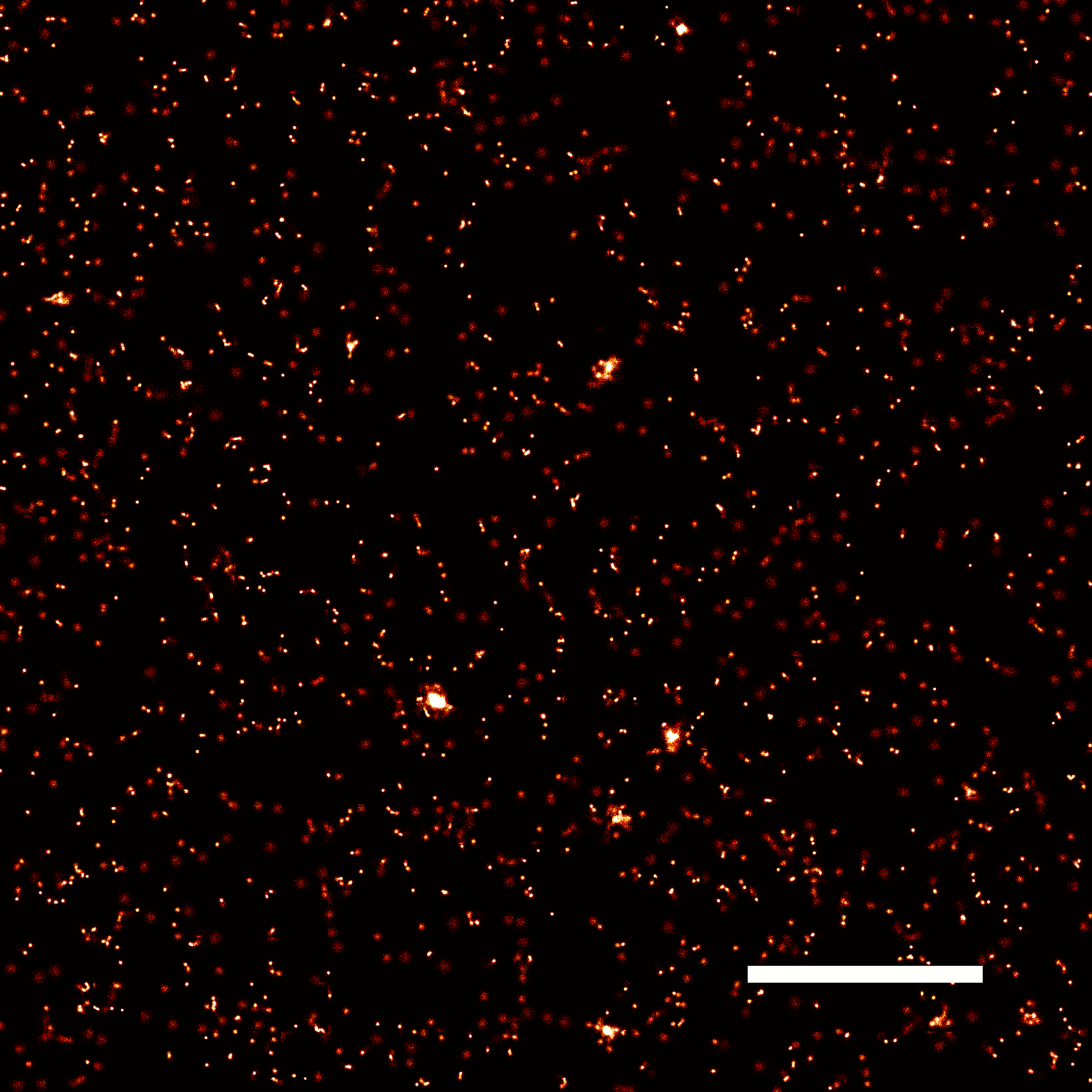

Supplement: Supplementary file 5 — Supplementary Software 1 [file 41467_2022_34894_MOESM5_ESM.zip › SoftwarePackage/Expected Results/Results_EGFR/Post-Im.png]

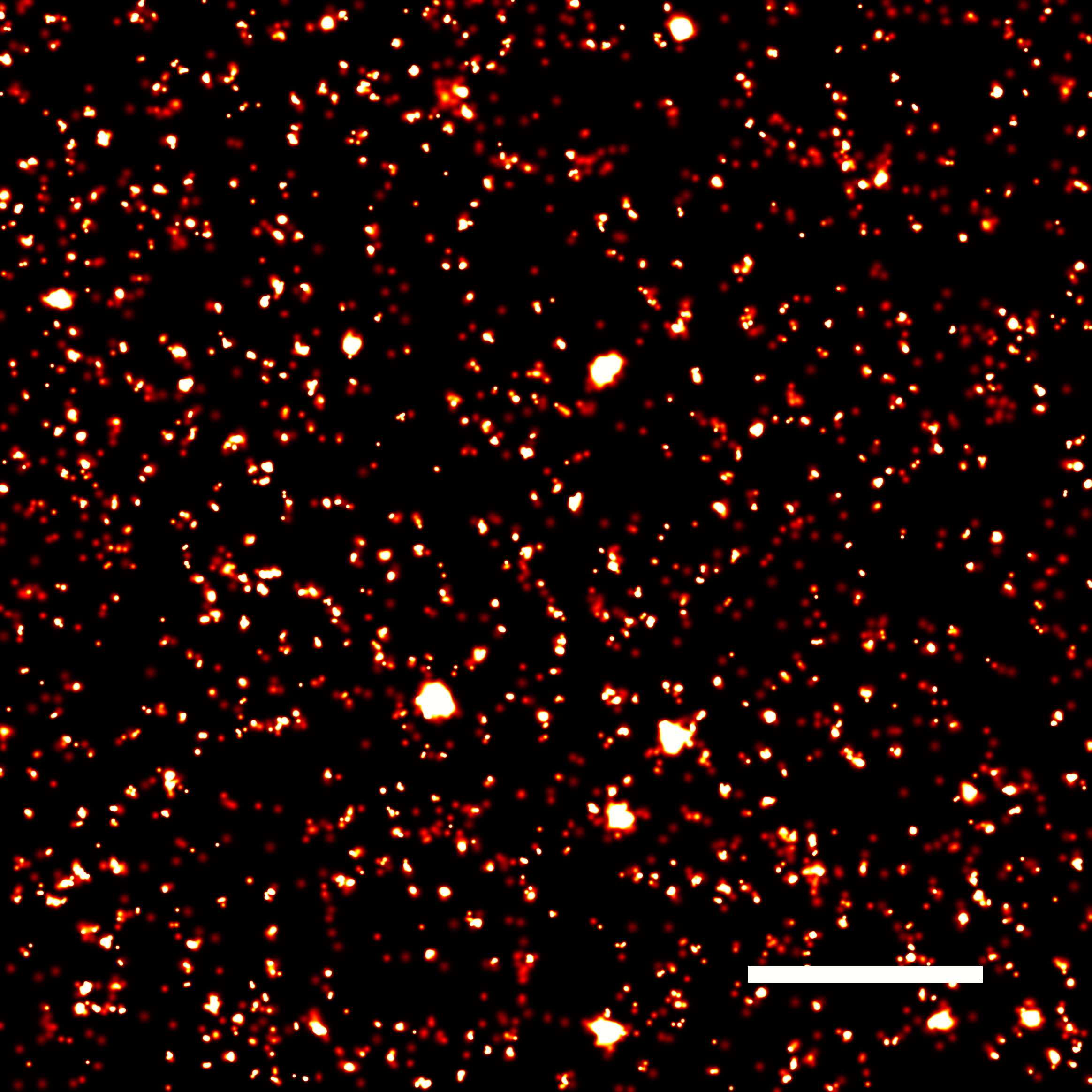

Supplement: Supplementary file 5 — Supplementary Software 1 [file 41467_2022_34894_MOESM5_ESM.zip › SoftwarePackage/Expected Results/Results_EGFR/SR-Im.png]

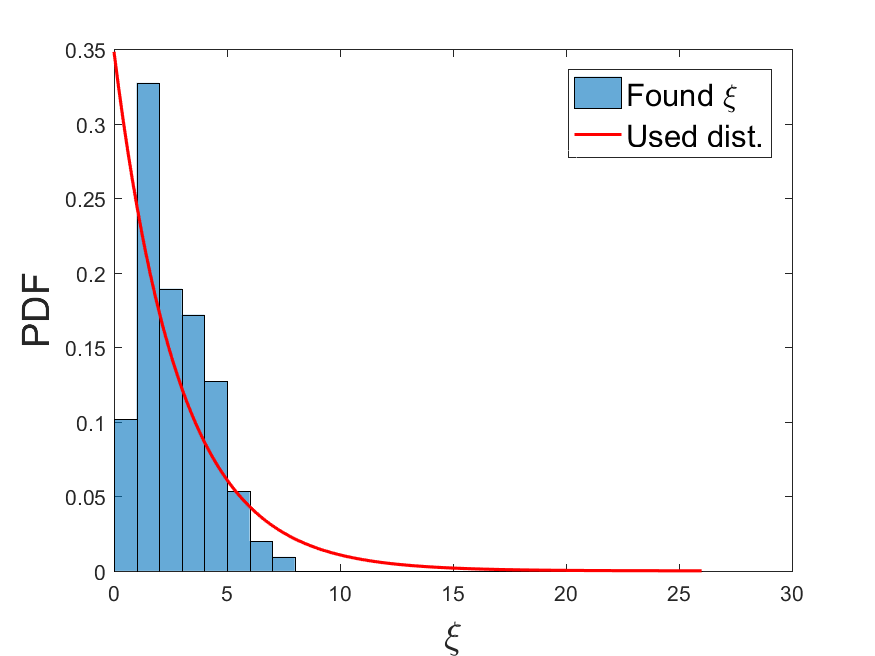

Supplement: Supplementary file 5 — Supplementary Software 1 [file 41467_2022_34894_MOESM5_ESM.zip › SoftwarePackage/Expected Results/Results_EGFR/Xi.png]

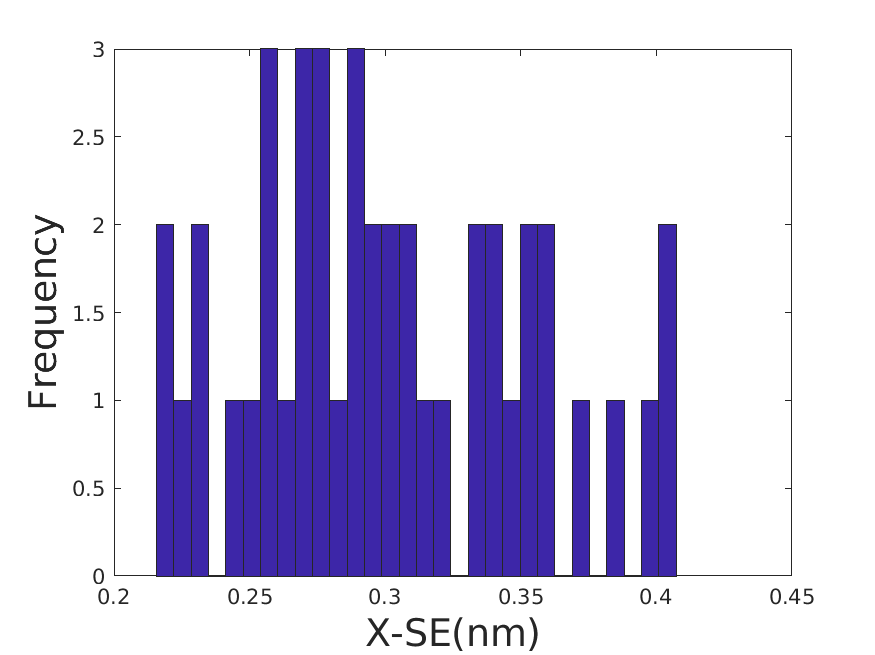

Supplement: Supplementary file 5 — Supplementary Software 1 [file 41467_2022_34894_MOESM5_ESM.zip › SoftwarePackage/Expected Results/Results_MPI/BaGoL_X-SE.png]

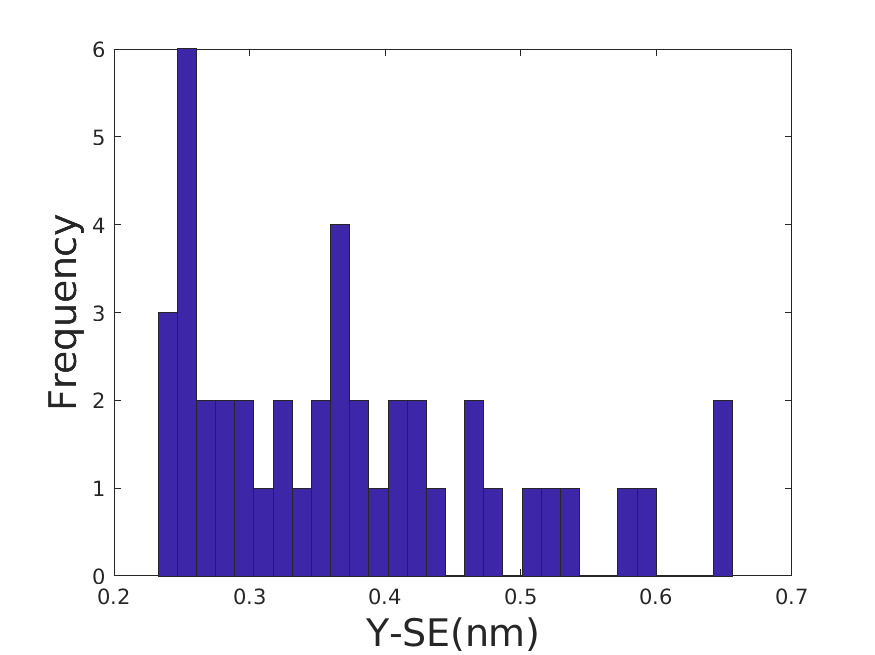

Supplement: Supplementary file 5 — Supplementary Software 1 [file 41467_2022_34894_MOESM5_ESM.zip › SoftwarePackage/Expected Results/Results_MPI/BaGoL_Y-SE.png]

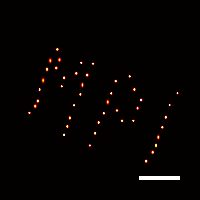

Supplement: Supplementary file 5 — Supplementary Software 1 [file 41467_2022_34894_MOESM5_ESM.zip › SoftwarePackage/Expected Results/Results_MPI/MAPN-Im.png]

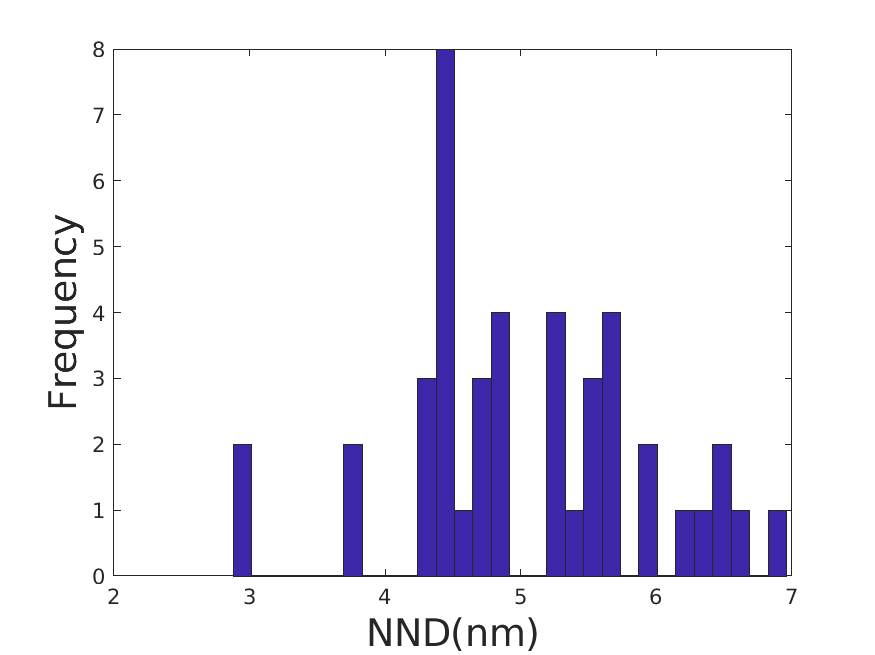

Supplement: Supplementary file 5 — Supplementary Software 1 [file 41467_2022_34894_MOESM5_ESM.zip › SoftwarePackage/Expected Results/Results_MPI/NND.png]

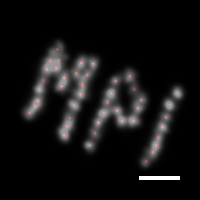

Supplement: Supplementary file 5 — Supplementary Software 1 [file 41467_2022_34894_MOESM5_ESM.zip › SoftwarePackage/Expected Results/Results_MPI/Overlay_SR_Map.png]

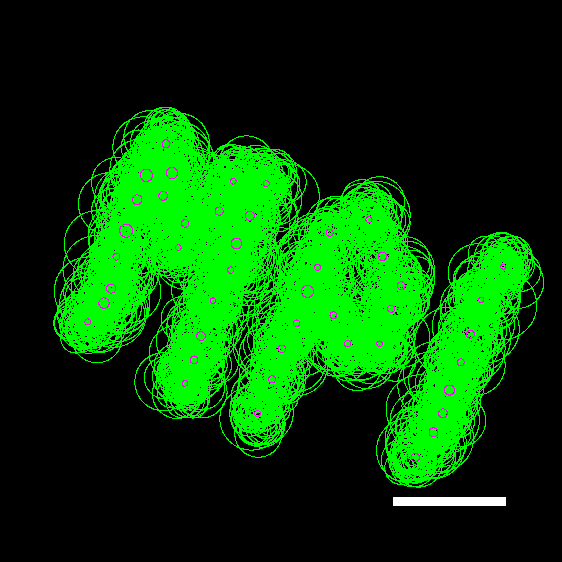

Supplement: Supplementary file 5 — Supplementary Software 1 [file 41467_2022_34894_MOESM5_ESM.zip › SoftwarePackage/Expected Results/Results_MPI/Overlay_SR_Map_circle.png]

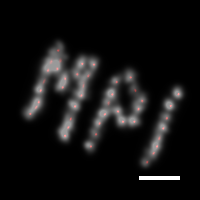

Supplement: Supplementary file 5 — Supplementary Software 1 [file 41467_2022_34894_MOESM5_ESM.zip › SoftwarePackage/Expected Results/Results_MPI/Overlay_SR_Post.png]

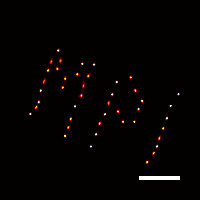

Supplement: Supplementary file 5 — Supplementary Software 1 [file 41467_2022_34894_MOESM5_ESM.zip › SoftwarePackage/Expected Results/Results_MPI/Post-Im.png]

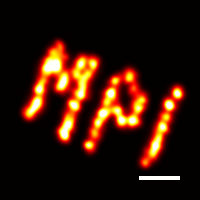

Supplement: Supplementary file 5 — Supplementary Software 1 [file 41467_2022_34894_MOESM5_ESM.zip › SoftwarePackage/Expected Results/Results_MPI/SR-Im.png]

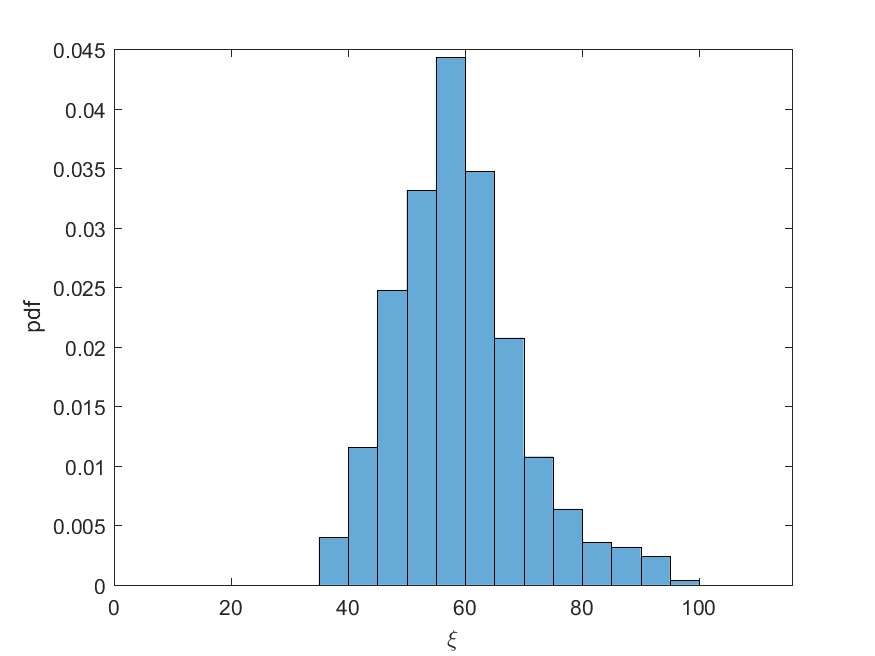

Supplement: Supplementary file 5 — Supplementary Software 1 [file 41467_2022_34894_MOESM5_ESM.zip › SoftwarePackage/Expected Results/Results_MPI/Xi.png]
