# Supplementary material for: High-precision estimation of emitter positions using Bayesian grouping of localizations
Source: Nat Commun. 2022 Nov 22;13:7152. doi: 10.1038/s41467-022-34894-2 (PMC9684143; doi:10.1038/s41467-022-34894-2)
Supplement: Supplementary file 6 — Reporting Summary [file 41467_2022_34894_MOESM6_ESM.pdf]

nature research

Corresponding author(s): Keith A. Lidke

Last updated by author(s): 10/31/2022

Reporting Summary

Nature Research wishes to improve the reproducibility of the work that we publish. This form provides structure for consistency and transparency in reporting. For further information on Nature Research policies, see our Editorial Policies and the Editorial Policy Checklist.

Statistics

For all statistical analyses, confirm that the following items are present in the figure legend, table legend, main text, or Methods section.

n/a	Confirmed	
☐	☒	The exact sample size (*n*) for each experimental group/condition, given as a discrete number and unit of measurement
☐	☒	A statement on whether measurements were taken from distinct samples or whether the same sample was measured repeatedly
☐	☒	The statistical test(s) used AND whether they are one- or two-sided<br>*Only common tests should be described solely by name; describe more complex techniques in the Methods section.*
☒	☐	A description of all covariates tested
☐	☒	A description of any assumptions or corrections, such as tests of normality and adjustment for multiple comparisons
☐	☒	A full description of the statistical parameters including central tendency (e.g. means) or other basic estimates (e.g. regression coefficient) AND variation (e.g. standard deviation) or associated estimates of uncertainty (e.g. confidence intervals)
☒	☐	For null hypothesis testing, the test statistic (e.g. *F*, *t*, *r*) with confidence intervals, effect sizes, degrees of freedom and *P* value noted<br>*Give P values as exact values whenever suitable.*
☐	☒	For Bayesian analysis, information on the choice of priors and Markov chain Monte Carlo settings
☐	☒	For hierarchical and complex designs, identification of the appropriate level for tests and full reporting of outcomes
☐	☒	Estimates of effect sizes (e.g. Cohen's *d*, Pearson's *r*), indicating how they were calculated

Our web collection on statistics for biologists contains articles on many of the points above.

Software and code

Policy information about availability of computer code

Data collection	DNA-ruler, EGFR, CD82 and microtubule data were acquired using the custom-written microscopy instrument control (MIC) package (https://github.com/LidkeLab/matlab-instrument-control).<br>DNA-origami data were collected using µManager (Edelstein, A.D. et al. J. Biol. Methods 1, 10 (2014)) a free open-source microscopy acquisition software.
Data analysis	DNA-ruler, EGFR, CD82 and microtubule data were localized prior to BaGoL using custom-written image analysis software developed based on Smith, et al, Nature Methods, 2010. (https://github.com/LidkeLab/smite)<br>DNA-origami data except MPI were localized using the BAMF algorithm described in Fazel, et al, Scientific Reports 2019.<br>MPI DNA-Origami data were analyzed prior to BaGoL using the PICASSO package, (Schnitzbauer, Nature Protocols, 2017).<br>All the BaGoL data analysis was performed using MATLAB 2020 (MathWorks Inc.).

For manuscripts utilizing custom algorithms or software that are central to the research but not yet described in published literature, software must be made available to editors and reviewers. We strongly encourage code deposition in a community repository (e.g. GitHub). See the Nature Research guidelines for submitting code & software for further information.

Data

Policy information about availability of data

All manuscripts must include a data availability statement. This statement should provide the following information, where applicable:

- Accession codes, unique identifiers, or web links for publicly available datasets
- A list of figures that have associated raw data
- A description of any restrictions on data availability

All the dSTORM data and DNA-PAINT data of Rulers, MPI, TUD and rectangles are included as supplimental files. All other data that supports the findings of this study are available from the corresponding author upon request.

Field-specific reporting

Please select the one below that is the best fit for your research. If you are not sure, read the appropriate sections before making your selection.

☒ Life sciences ☐ Behavioural & social sciences ☐ Ecological, evolutionary & environmental sciences

For a reference copy of the document with all sections, see nature.com/documents/nr-reporting-summary-flat.pdf

Life sciences study design

All studies must disclose on these points even when the disclosure is negative.

Sample size	This manuscript is a technology development method. The method was tested extensively with simulations, whereas biological demonstrations were limited to measurements of single cells of various types and structures. We did not perform any sample size calculations.
Data exclusions	No data was excluded.
Replication	Details of data simulation, collection and analysis used in this study is provided in the methods section.
Randomization	No randomization was used in this study.
Blinding	No blinding was used in this study.

Reporting for specific materials, systems and methods

We require information from authors about some types of materials, experimental systems and methods used in many studies. Here, indicate whether each material, system or method listed is relevant to your study. If you are not sure if a list item applies to your research, read the appropriate section before selecting a response.

Materials & experimental systems

n/a	Involved in the study
☐	☒ Antibodies
☐	☒ Eukaryotic cell lines
☒	☐ Palaeontology and archaeology
☒	☐ Animals and other organisms
☒	☐ Human research participants
☒	☐ Clinical data
☒	☐ Dual use research of concern

Methods

n/a	Involved in the study
☒	☐ ChIP-seq
☒	☐ Flow cytometry
☒	☐ MRI-based neuroimaging

Antibodies

Antibodies used	Alexa Fluor 647 anti–human CD82 antibody; BioLegend, ASL-24, lot number: B262939<br>fluorescently-labeled EGF: Purchased from ThermoFisher (Thermo Fisher Scientific, #E35351)<br>anti-GFP Nanobody: Purchased from NanoTag Biotechnologies (FluoTag-Q anti-GFP, unconjugated, C-terminal ectopic cysteine).<br>alpha-tubulin: Purchased from NOVUS Biologicals, part # NB100-690AF647
Validation	Alexa Fluor 647 anti–human CD82 antibody: Each lot of this antibody is quality control tested by immunofluorescent staining with flow cytometric analysis by Biolegend. According to the manufacturer, the antibody was purified by affinity chromatography and conjugated with Alexa Fluor® 647 under optimal conditions. The solution is free of unconjugated Alexa Fluor® 647. This product lot has passed BioLegend's QC testing and is certified for use. For details on QC testing view our page at biolegend.com/en-us/quality-control.

April 2020

fluorescently-labeled EGF: validated by 1) it does not bind cells that do not express EGFR; and 2) binding of AF647-EGF to EGFR induces receptor phosphorylation (by western blot) and endocytosis (microscopy).

anti-GFP Nanobody: Visibly bound to correct structure expressing GFP. Böttcher RT, et al. The Journal of cell biology, 216(11):3785–3798.

alpha-tubulin: Visibly bound to correct structure.

Eukaryotic cell lines

Policy information about cell lines

Cell line source(s)	CHO cells expressing EGFR-GFP were originally generated by the Jovin group (MPI PMC, Germany: Lidke et al NBT 2004).
Authentication	HEK 293 and HeLa cells were confirmed by STR analysis (ATCC).<br>CHO cells were routinely checked for EGFR receptor proper expression levels and function.
Mycoplasma contamination	Cells were confirmed to by free from mycoplasma using either MycoAlert Mycoplasma Detection Kit (Lonza) or MycoStrip Mycoplasma Detection Kit (InvivoGen).
Commonly misidentified lines<br>(See ICLAC register)	*Name any commonly misidentified cell lines used in the study and provide a rationale for their use.*
